# In vivo characterization of the activities of novel cyclodipeptide oxidases: new tools for increasing chemical diversity of bioproduced 2,5-diketopiperazines in *Escherichia coli*

**DOI:** 10.1186/s12934-020-01432-y

**Published:** 2020-09-07

**Authors:** Fabien Le Chevalier, Isabelle Correia, Lucrèce Matheron, Morgan Babin, Mireille Moutiez, Nicolas Canu, Muriel Gondry, Olivier Lequin, Pascal Belin

**Affiliations:** 1 Université Paris-Saclay, CEA, CNRS, Institute for Integrative Biology of the Cell (I2BC), 91198 Gif-sur-Yvette, France; 2 Sorbonne Université, Institut de Biologie Paris Seine (IBPS), FRE3631, 75005 Paris, France; 3 Laboratoire des Biomolécules (LBM), Sorbonne Université, Ecole Normale Supérieure, PSL University, CNRS, 75005 Paris, France

**Keywords:** Synthetic biology, Combinatorial biosynthesis, Natural products, 2,5-diketopiperazine, Cyclodipeptide synthase, Cyclodipeptide oxidase

## Abstract

**Background:**

Cyclodipeptide oxidases (CDOs) are enzymes involved in the biosynthesis of 2,5-diketopiperazines, a class of naturally occurring compounds with a large range of pharmaceutical activities. CDOs belong to cyclodipeptide synthase (CDPS)-dependent pathways, in which they play an early role in the chemical diversification of cyclodipeptides by introducing Cα-Cβ dehydrogenations. Although the activities of more than 100 CDPSs have been determined, the activities of only a few CDOs have been characterized. Furthermore, the assessment of the CDO activities on chemically-synthesized cyclodipeptides has shown these enzymes to be relatively promiscuous, making them interesting tools for cyclodipeptide chemical diversification. The purpose of this study is to provide the first completely microbial toolkit for the efficient bioproduction of a variety of dehydrogenated 2,5-diketopiperazines.

**Results:**

We mined genomes for CDOs encoded in biosynthetic gene clusters of CDPS-dependent pathways and selected several for characterization. We co-expressed each with their associated CDPS in the pathway using *Escherichia coli* as a chassis and showed that the cyclodipeptides and the dehydrogenated derivatives were produced in the culture supernatants. We determined the biological activities of the six novel CDOs by solving the chemical structures of the biologically produced dehydrogenated cyclodipeptides. Then, we assessed the six novel CDOs plus two previously characterized CDOs in combinatorial engineering experiments in *E. coli*. We co-expressed each of the eight CDOs with each of 18 CDPSs selected for the diversity of cyclodipeptides they synthesize. We detected more than 50 dehydrogenated cyclodipeptides and determined the best CDPS/CDO combinations to optimize the production of 23.

**Conclusions:**

Our study establishes the usefulness of CDPS and CDO for the bioproduction of dehydrogenated cyclodipeptides. It constitutes the first step toward the bioproduction of more complex and diverse 2,5-diketopiperazines.

## Background

2,5-diketopiperazines (2,5-DKPs) are a large class of molecules characterized by a 2,5-DKP ring, resulting from the condensation of two α-amino acids [1]. Originally isolated from natural products more than 100 years ago, they have then been largely studied and developed in medicinal chemistry for their noteworthy biological activities [1]. 2,5-DKPs exhibit a large spectrum of chemical structures, ranging from simple cyclic dipeptides (CDPs) to polycyclic compounds carrying various chemical modifications. Their biosynthesis has been explored recently with the discovery of dedicated biosynthetic pathways, revealing the diversity of enzymes involved in the assembly and the tailoring of the CDP core [2–5]. In the context of synthetic biology, the characterization and the exploitation of enzymes of the 2,5-DKP biosynthetic pathways appear to be an efficient means to access a novel chemical diversity of potentially bioactive 2,5-DKPs [6].

**Fig. 1:**
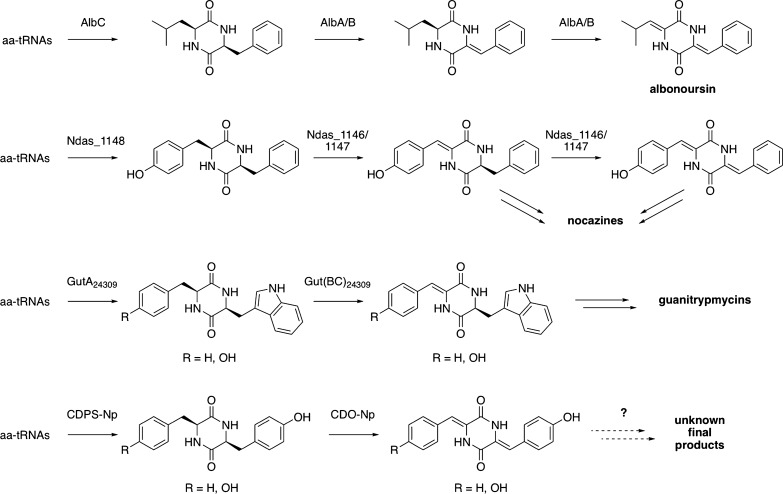
Activities of CDPSs and CDOs in biosynthetic pathways. The names of the final products of the pathways are indicated. Dashed arrows indicate hypothetical subsequent tailoring steps in the biosynthetic pathway. The activities of PcmA (CDPS) and PcmB/C (CDO) involved in purincyclamide biosynthesis are similar to those of GutA_24309_ and Gut(BC)_24309_, respectively, and purincyclamide is identical to guanitrypmycin A2-1 [11, 12]

Cyclic dipeptide oxidases (CDOs) are tailoring enzymes found in 2,5-DKP biosynthetic pathways that depend on cyclodipeptide synthases (CDPSs) [2, 7]. CDPSs catalyse the first step of these pathways by using aminoacyl-tRNAs (aa-tRNAs) to synthesize CDPs. Then tailoring enzymes introduce chemical modifications into the CDPs, including the dehydrogenation of the Cα-Cβ bonds catalysed by CDOs [2, 4, 8]. Four CDO-containing pathways have been unravelled, revealing the activities of the corresponding CDOs: AlbA/B of the albonoursin biosynthetic pathway from *Streptomyces noursei*, Ndas_1146/1147 of the nocazines biosynthetic pathway from *Nocardiopsis dassonvillei*, Gut(BC)_24309_ of the guanitrypmycins biosynthetic pathway from *Streptomyces monomycini*, and PcmB/C of the purincyclamide biosynthetic pathway from *Streptomyces chrestomyceticus* [9–12] (Fig. 1). Furthermore, incomplete data have been obtained for a fifth CDO-containing pathway encoded in *Nocardiopsis prasina*, with CDPS-Np synthesizing cYF and cYY and CDO-Np catalysing the tetra-dehydrogenation of these CDPs [13, 14]. CDOs are comprised of two subunits of approximately 21 and 11 kDa, referred to hereafter as CDOA and CDOB, respectively. CDOA and CDOB are encoded by two genes that overlap by 20-30 nucleotides, a feature believed to be important for CDO production as attempts to produce recombinant CDO from two independent monocistronic operons have systematically failed [9, 10]. CDOA, CDOB, and a flavinic cofactor assemble into an active high molecular weight heteropolymeric CDO that uses molecular oxygen to catalyse CDP dehydrogenation [10, 15].

Studies focusing on CDO specificity and the utilization of CDOs as 2,5-DKP tailoring enzymes are scarce. Preliminary studies on CDO specificity showed that they can accept CDPs other than those synthesized by their associated CDPSs, with the CDPs having an aromatic side chain being better substrates [15–17]. Additionally, the group of Kanzaki reported the conversion of phenylahistine, a prenylated CDP, to its dehydro- derivative by the CDO from *Streptomyces albulus* KO-23, showing the acceptance of more complex CDP substrates by CDOs [18]. Recently, AlbA/B, Ndas_1146/1147, and CDO-Np were investigated for the conversion of a large set of chemically synthesized cyclodipeptides and analogues [14]. The authors used either bioconversion using recombinant *Escherichia coli* bacteria that overproduce CDO or in vitro conversion using a bacterial crude extract containing recombinant CDO. The CDOs exhibited a strong preference for CDP containing l-Phe or l-Tyr. Compounds with a benzodiazepine di-one ring instead of the 2,5-DKP ring were not converted, suggesting that the 2,5-DKP ring may be an important substrate determinant for CDO activity. This recent study also showed differences in specificity between CDOs, highlighting the interest of having a wide variety of CDOs for modifying a large range of CDPs. Thus, investigating the activity of novel CDOs should provide new tools for CDP chemical diversification, while allowing a better understanding of the biosynthetic pathways the CDOs are associated with.

Over the last 10 years, the number of CDPSs has exploded, whether identified in genomes as putative enzymes or biochemically characterized. Several hundreds non-redundant putative CDPSs have been identified in genomes [5, 8, 19, 20], and the activities of approximately 120 CDPSs have been described by following the appearance of CDPs secreted into the culture supernatants of *E. coli* bacteria overexpressing a CDPS [10, 13, 17, 19, 21–27]. A global analysis of 721 cyclodipeptide biosynthetic gene clusters showed the association of a CDPS gene with CDO genes in 42 of them [20]. Furthermore, many of these associations are original in terms of the biosynthetic gene cluster composition or the activity of the associated CDPS, suggesting novel activities for the corresponding CDOs. The straightforward production of active CDPSs and CDOs in *E. coli* led us to implement a completely recombinant approach to characterize the activity of novel CDOs. Here, we describe the bioinformatic analysis of the CDO subunits, the characterization of the activities of seven previously undescribed CDOs in *E. coli*, and the evaluation of their usefulness for the bioconversion of various CDPs produced by CDPSs in combinatorial approaches using *E. coli* as a host organism.

## Results

### Bioinformatic analysis of putative CDO subunits encoded in genomes

**Fig. 2:**
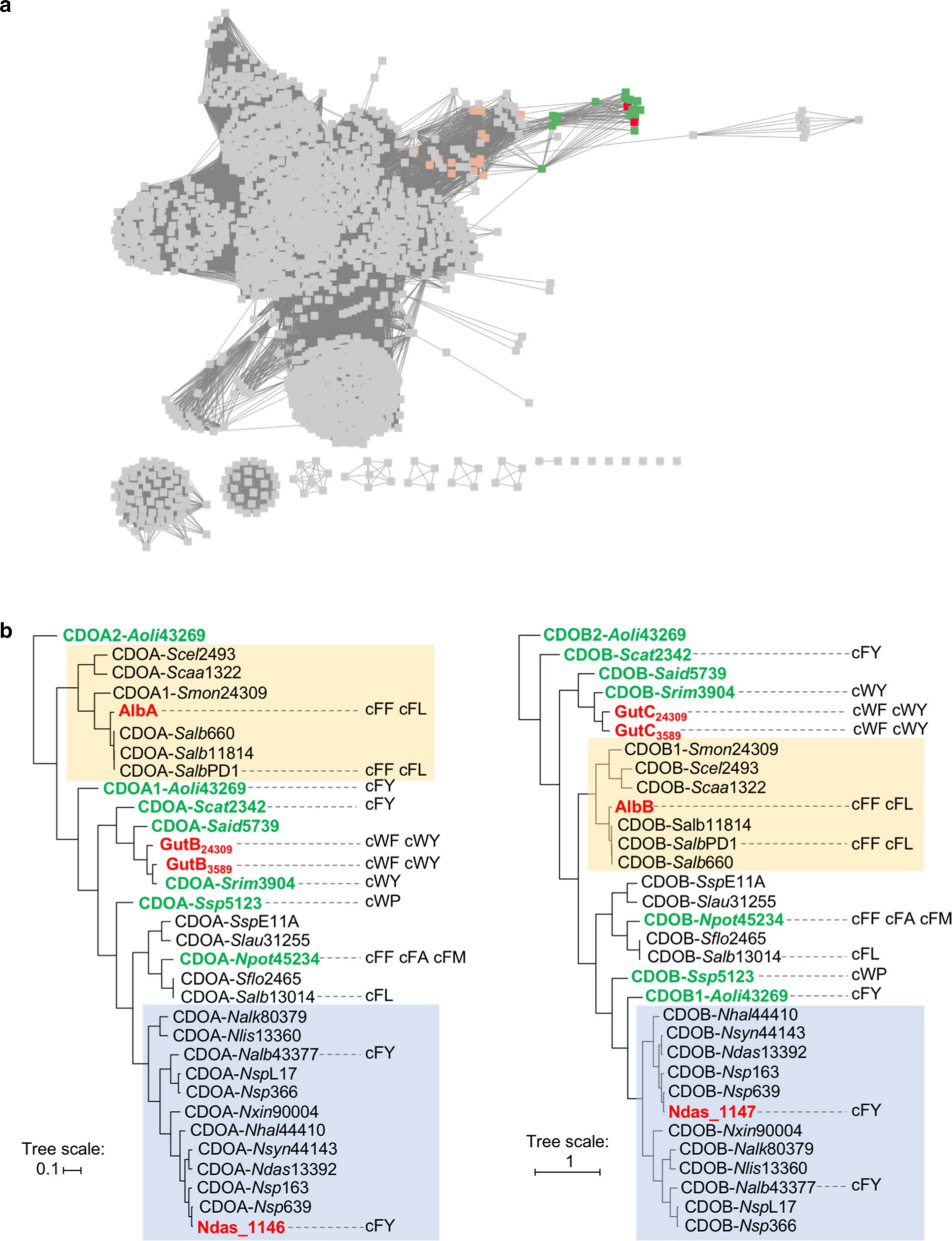
Diversity of CDOs. **a** A sequence similarity network was constructed with 1610 sequences homologous to CDOA-*Snou*11455, which are represented as nodes. Edges between nodes indicate sequence identity > 26.5%. The nodes corresponding to CDOA-*Snou*11455 and CDOA-*Ndas*43111 are shown in red. Nodes corresponding to CDOA subunits, for which the gene is associated in the genome with *cdoB* and *cdps* genes are shown in green. Salmon-coloured nodes correspond to CDOA subunits linked by an edge to CDOA subunits coloured in green or red. **b** Phylogenetic tree of 32 selected sequences of CDOA subunits (left panel) and CDOB subunits (right panel). The main product of the activity of the CDPS associated with the corresponding CDO in the biosynthetic gene cluster is indicated as cXX, X being one l-amino acid. Subunit names of CDOs of *S. noursei* ATCC 11455, *S. monomycini* NRRL B‐24309, *S. varsoviensis* NRRL B‐3589 and *N. dassonvillei* DSM 43111 are written in red. Clades encompassing subunits of CDOs of *S. noursei* ATCC 11455 and *N. dassonvillei* DSM 43111 are highlighted by rectangles coloured in yellow and blue, respectively. Subunit names of CDOs investigated in this study are coloured in green

We used bioinformatic tools at the Enzyme Function Initiative website to obtain a global view of CDOs encoded in genomes. We collected 1610 protein sequences homologous to CDOA-*Snou*11455 (AlbA) from the UniProt database and generated a protein sequence similarity network [28] (SSN; Fig. 2a). CDOA-*Snou*11455 and CDOA-*Ndas*43111 (CDOA subunits of the two most-studied CDOs) are clustered with a first set of CDOA sequences sharing over 52% sequence identity (nodes coloured in green in Fig. 2a). Examination of the genomic environment of the corresponding *cdoA* genes showed the association of CDOB- and CDPS-encoding genes. The SSN also displays a second set of sequences linked to the previously identified CDOA proteins by their edges (nodes coloured in salmon in Fig. 2a). Percentage identity between sequences of the first and second set are between 30 and 42%. Inspection of the genomic environment of the genes encoding sequences of this second set revealed the presence of a *cdoB* gene, but the absence of an associated CDPS gene. We continued our study focusing on the first set of CDOA sequences.

Using CDOA-*Snou*11455 and CDOA-*Ndas*43111 sequences as an entry point, we collected 30 additional CDOA sequences from the NCBI database, together with their associated CDOB sequences, thus constituting two sets of 32 sequences (Additional file 1: Table S1). Phylogenetic trees of CDOA and CDOB sequences were built using tools of the EMBL-EBI website (Fig. 2b). The sequences of subunits of CDO-*Snou*11455 and CDO-*Ndas*43111 are located within two clades (Fig. 2b). To expand the diversity of studied CDOs, we selected CDOs for which the subunit sequences are located outside these two clades (Fig. 2b).

### Co-expression of putative CDOs with their associated CDPSs results in the production of dehydrogenated cyclodipeptides

At the beginning of this study, two of the selected CDOs, CDO2-*Aoli*43269 and CDO-*Said*5739, were found in biosynthetic gene cluster (BGC) with CDPSs of unknown activity, CDPS2-*Aoli*43269 and CDPS-*Said*5739 (Additional file 1: Table S2). We investigated the activity of these two CDPSs as previously reported [19, 23]. CDPS2-*Aoli*43269 synthesized exclusively cWW. Three different cyclodipeptides were detected in culture supernatants of bacteria overexpressing CDPS-*Said*5739, the most abundant being cWL (97% according to its peak area on the chromatogram recorded at 214 nm), the other cyclodipeptides being cWF (3%), and cFL (detected only by ionic current). This is consistent with a recent characterization of the activity of DmtB3 of *S. aidingensis* (100% identical to CDPS-*Said*5739) [29].

**Fig. 3:**
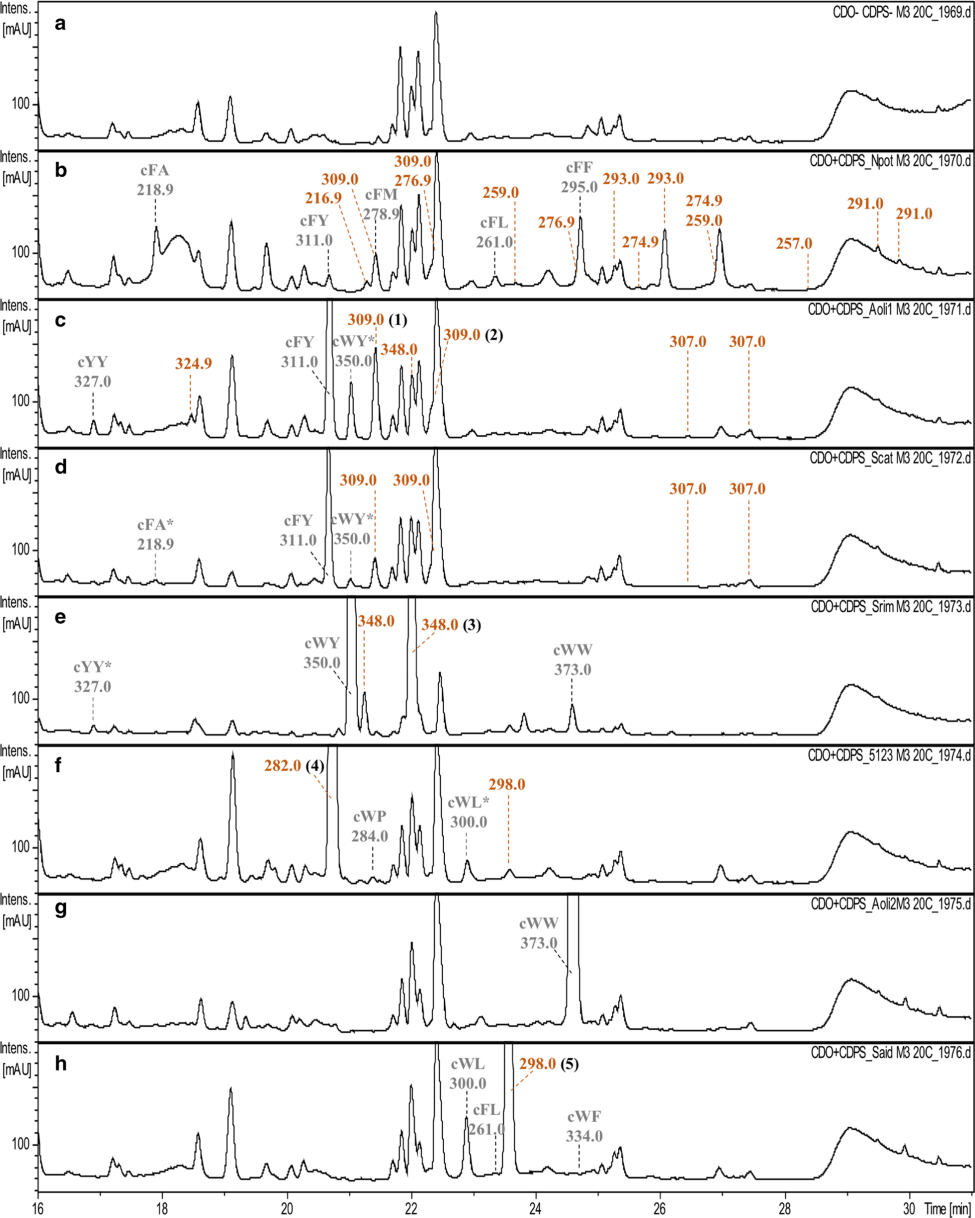
Detection of CDPs and their derivatives in culture supernatants of *E. coli* bacteria overexpressing CDPS and CDO. Culture supernatants of *E. coli* bacteria overexpressing no enzyme (**a**) or a CDPS/CDO couple from one pathway (**b**, *N. potens*; **c**, pathway 1 of *A. oligospora*; **d**, *S. catenulae*; **e**, *S. rimosus*; **f**, *S.* sp. NRRL 5123; **g**, pathway 2 of *A. oligospora*; **h**, *S. aidingensis*) were collected, treated by SPE, and analysed by LC–MSMS. UV traces recorded at 214 nm are shown between 16 and 31 min. The Y axes of the UV_214_ traces were set from 20 to 280 mAU. Recovered CDPs and the *m*/*z* values of their MH^+^ ions are indicated in grey. Stars indicate CDPs that have not been previously described for the characterized CDPSs. The *m*/*z* values of detected MH^+^ parent ions corresponding to dehydrogenated CDPs (loss of 2 and 4 atomic mass units) are shown in brown. The experiment was performed three times with similar results. Compounds chosen for purification and NMR characterization are indicated in black bold Arabic numbers in brackets (**1**–**5**)

We characterized the activities of the novel CDOs by co-expressing the CDPS and CDO genes from the same BGC in *E. coli* and examining the resulting 2,5-DKPs in SPE-treated culture supernatants by LC–MS/MS (Fig. 3). The five main CDPs produced by CDPS-*Npot*45234 (cFA, cFM, cFY, cFL, and cFF) were recovered and compounds with *m*/*z* values corresponding to dehydrogenated CDPs were also detected (Fig. 3b). The predicted ∆cFF (*m*/*z* 293, RT = 26.1 min) was the most abundant, shown by the corresponding peak on the UV chromatogram. Most of the other predicted dehydrogenated CDPs were produced in very low amounts. CDPS1-*Aoli*43269 and CDPS-*Scat*2342 produced mainly cYF (Fig. 3c, d). The co-expression of the cognate CDO led to the detection of two compounds predicted to be ∆cYF (*m*/*z* 309) in both cases, with RTs of 21.4 and 22.3 min, respectively. The compounds characterized by a similar RT in each sample displayed similar fragmentation patterns (Additional file 2: Fig. S1). The co-expression of CDPS and CDO of *S. rimosus* led to cWY and two compounds corresponding to the predicted di-dehydrogenated CDPs at *m*/*z* 348 (Fig. 3e). The main product of the CDPS-*S*sp5123 activity, cWP, was observed in low amounts and a large peak containing the predicted ∆cWP at *m*/*z* 282 was observed on the UV chromatogram (Fig. 3f). The co-expression of the enzymes of *S. aidingensis* led to a large peak on the UV chromatogram at *m*/*z* 298, corresponding to the predicted ∆cWL, and the CDP cWL at *m*/*z* 300 corresponding to a smaller peak (Fig. 3h). Finally, we detected no predicted dehydrogenated CDPs in samples resulting from the co-expression of CDPS2 and CDO2 of *A. oligospora*, although cWW, the product of CDPS2-*Aoli*43269 activity, was clearly produced in high amounts (Fig. 3g).

We performed LC-HRMS analysis of the SPE-treated culture supernatants to obtain a more accurate mass of the predicted dehydrogenated products. The 2,5-DKPs detected upon co-expression of a CDPS and a CDO originating from the same pathway are summarized in Table 1. The potential dehydrogenated CDPs recovered by LC-HRMS were measured with high mass accuracy (∆*m*/*z* ranged from 0 to 0.86 ppm of the [M + H]^+^ parent ions) and precision (two independent experiments, each injected twice). Thus, the LC-HRMS data confirmed the presence of dehydrogenated CDPs suggested by the LC–MS/MS data, except for the compound at *m*/*z* 348 (predicted ∆cWY) produced by the combination of CDPS1-*Aoli*43269 and CDO1-*Aoli*43269 (Fig. 3c). Some of the other differences between the LC–MS/MS and LC-HRMS data are related to the number of isoforms found for one dehydrogenated CDP (in particular for ∆cFY).

**Table 1:** LC-HRMS analysis of SPE-treated supernatants of bacterial cultures expressing a CDPS/CDO couple originating from the same operon

Host of origin	Searched compound					
	Name	Molecular formula of [M + H]^+^	Expected m/z	Observed m/z	RT (min)^a^	Peak area (EIC)
*Nocardiopsis potens*	cFA	C_12_H_15_N_2_0_2_^+^	219.1128	219.1127	32.75	8.33E+05
	∆cFA	C_12_H_13_N_2_0_2_^+^	217.0972	217.0971	33.57	3.49E+04
				217.0971	38.58	3.04E+05
	∆∆cFA	C_12_H_11_N_2_0_7_^+^	215.0815	–	–	–
	cFY	C_18_H_19_N_2_0_3_^+^	311.1390	311.1390	37.41	4.43E+05
	∆cFY	C_18_H_17_N_2_0_3_^+^	309.1234	309.1234	38.75	3.34E+05
				309.1234	39.57	4.58E+05
	∆∆cFY	C_18_H_15_N_2_0_3_^+^	307.1077	307.1077	44.02	6.13E+04
	cFM	C_14_H_19_N_2_0_2_S^+^	279.1162	279.1162	39.11	3.92E+06
	∆cFM	C_14_H_17_N_2_0_2_S^+^	277.1005	277.1006	40.08	7.76E+05
				277.1006	42.16	7.75E+05
	∆∆cFM	C_14_H_15_N_2_0_2_S^+^	275.0849	275.0849	43.17	3.86E+04
				275.0849	44.05	1.59E+05
	cFL	C_15_H_21_N_2_0_2_^+^	261.1598	261.1598	40.93	1.36E+05
	∆cFL	C_15_H_19_N_2_0_2_^+^	259.1441	259.1441	41.36	2.17E+05
				259.1441	44.26	5.70E+05
	∆∆cFL	C_15_H_17_N_2_0_2_^+^	257.1285	257.1284	44.79	1.59E+04
				257.1284	45.75	9.23E+04
	cFF	C_18_H_19_N_2_0_2_^+^	295.1441	295.1441	41.81	6.13E+06
	∆cFF	C_18_H_17_N_2_0_2_^+^	293.1285	293.1285	42.58	4.09E+05
				293.1284	43.17	7.77E+06
	∆∆cFF	C_18_H_15_N_2_0_2_^+^	291.1128	291.1128	46.98	1.62E+05
				291.1128	47.9	2.55E+05
*Actinomadura oligospora* (pathway 1)	cFY	C_18_H_19_N_2_0_3_^+^	311.1390	311.1390	37.62	1.01E+07
	∆cFY	C_18_H_17_N_2_0_3_^+^	309.1234	309.1233	37.17	1.37E+05
				309.1234	38.67	4.09E+06
				309.1233	39.78	2.26E+06
				309.1233	40.14	2.00E+05
	∆∆cFY	C_18_H_15_N_2_0_3_^+^	307.1077	307.1077	43.6	8.98E+04
				307.1078	44.49	4.63E+05
	cYY	C_18_H_19_N_2_0_4_^+^	327.1339	327.1339	29.92	1.25E+05
	∆cYY	C_18_H_17_N_2_0_4_^+^	325.1183	325.1183	32.60	2.21E+05
	∆∆cYY	C_18_H_15_N_2_0_4_^+^	323.1026	323.1028	41.48	5.52E+04
	cWY	C_20_H_20_N_3_0_3_^+^	350.1499	350.1499	37.65	4.37E+05
	∆cWY	C_20_H_18_N_3_0_3_^+^	348.1343	–	–	–
	∆∆cWY	C_20_H_16_N_3_0_3_^+^	346.1186	–	–	–
*Streptomyces catenulae*	cFY	C_18_H_19_N_2_0_3_^+^	311.1390	311.1390	37.51	9.80E+06
	∆cFY	C_18_H_17_N_2_0_3_^+^	309.1234	309.1233	37.18	9.64E+04
				309.1234	38.76	1.89E+06
				309.1234	39.59	3.13E+06
				309.1234	39.97	1.81E+05
	∆∆cFY	C_18_H_15_N_2_0_3_^+^	307.1077	307.1077	42.82	1.74E+04
				307.1076	43.64	5.59E+04
	cWY	C_20_H_20_N_3_0_3_^+^	350.1499	350.1499	37.66	3.41E+05
	∆cWY	C_20_H_18_N_3_0_3_^+^	348.1343	–	–	–
	∆∆cWY	C_20_H_16_N_3_0_3_^+^	346.1186	–	–	–
*Streptomyces rimosus*	cWY	C_20_H_20_N_3_0_3_^+^	350.1499	350.1498	37.40	1.01E+07
	∆cWY	C_20_H_18_N_3_0_3_^+^	348.1343	348.1343	38.41	1.81E+06
				348.1340	38.73	9.73E+06
				348.1342	39.02	2.45E+06
	∆∆cWY^b^	C_20_H_16_N_3_0_3_^+^	346.1186	–	–	–
	cWW	C_22_H_21_N_4_0_2_^+^	373.1659	373.1659	41.62	2.71E+06
	∆cWW^b^	C_22_H_19_N_4_0_2_^+^	371.1503	–	–	–
	∆∆cWW	C_22_H_17_N_4_0_2_^+^	369.1346	–	–	–
*Streptomyces* sp. NRRL F–5123	cWP	C_16_H_18_N_3_0_2_^+^	284.1394	284.1393	38.08	1.80E+05
	∆cWP	C_16_H_16_N_3_0_2_^+^	282.1237	282.1235	37.33	1.80E+07
	∆cWP	C_16_H_14_N_3_0_2_^+^	280.1081	280.1081	37.36	1.04E+05
	cWL	C_17_H_22_N_3_0_2_^+^	300.1707	300.1706	39.8	1.19E+06
	∆cWL	C_17_H_20_N_3_0_2_^+^	298.1550	298.1550	40.64	7.38E+05
	∆∆cWL	C_17_H_18_N_3_0_2_^+^	296.1394	–	–	–
*Actinomadura oligospora* (pathway 2)	cWW	C_22_H_21_N_4_0_2_^+^	373.1659	373.1658	41.04	1.28E+07
	∆cWW	C_22_H_19_N_4_0_2_^+^	371.1503	371.1504	41.18	1.50E+05
	∆∆cWW	C_22_H_17_N_4_0_2_^+^	369.1346	369.1345	41.15	1.00E+04
*Streptomyces aidingensis*	cWL	C_17_H_22_N_3_0_2_^+^	300.1707	300.1706	40.06	3.26E+06
	∆cWL	C_17_H_20_N_3_0_2_^+^	298.1550	298.1550	40.95	1.28E+07
	∆∆cWL	C_17_H_18_N_3_0_2_^+^	296.1394	–	–	–
	cFL	C_15_H_21_N_2_0_2_^+^	261.1598	261.1598	41.18	7.00E+04
	∆cFL	C_15_H_19_N_2_0_2_^+^	259.1441	–	–	–
	∆∆cFL	C_15_H_17_N_2_0_2_^+^	257.1285	–	–	–
	cWF	C_20_H_20_N_3_0_2_^+^	334.1550	334.1550	41.65	1.29E+05
	∆cWF	C_20_H_18_N_3_0_2_^+^	332.1394	332.1393	42.66	1.96E+05
	∆∆cWL	C_20_H_16_N_3_0_2_^+^	330.1237	–	–	–

Experiments from bacterial transformations to LC-HRMS were performed twice independently and each sample was injected twice on an LC-HRMS; one set of results (one experiment, one injection) is presented; differences between samples corresponding to the same experimental condition are indicated

^a^RT, retention time

^b^Trace amounts were detected in some analysis

Altogether, these results show that all but one studied CDOs (CDO2-*Aoli*43269 excepted) catalyse the efficient dehydrogenation of the major cyclodipeptide(s) synthesized by the associated CDPS in a BGC, albeit with different efficiencies.

### Structural characterizations of bio-produced 2,5-DKPs reveal dehydrogenation site preferences for CDOs

We bio-produced and purified five predicted dehydrogenated CDPs (compounds **1**–**5** in Fig. 3). We structurally characterized them by ^1^H, ^13^C, and ^15^N NMR spectroscopy (Additional file 1: Tables S3–S4 and Additional file 2: Fig. S2–S11). The analysis of 2D ^1^H, ^13^C heteronuclear multiple bond correlation (HMBC) experiments enabled us to identify the site of desaturation of di-dehydrogenated CDPs. Furthermore, the *Z* configuration of the double bond could be unambiguously established through the measurement of ^3^*J* (^1^H, ^13^C) coupling constants, showing a *cis* relationship between the alkene proton and the carbonyl group, together with the observation of through space ROE correlations between the amide proton and side-chain protons of the desaturated amino acid (Additional file 1: Table S4).

The compounds **1** and **2** resulted from the co-expression of CDPS1-*Aoli*43269 and CDO1-*Aoli*43269 and were predicted derivatives of cYF (Fig. 3c). NMR data showed that **1** corresponds to the *Z* isomer of cY∆F and **2** the *Z* isomer of c∆YF (Additional file 1: Tables S3–S4 and Additional file 2: Fig. S2–S5). Two predicted di-dehydrogenated cWY were observed upon co-expression of CDPS-*Srim*3904 and CDO-*Srim*3904 (Fig. 3e). We purified **3**, but did not succeed in purifying the other compound with a [M + H]^+^ parent ion at *m*/*z* 348. NMR data indicated that **3** is the *Z* isomer of cW∆Y (Additional file 1: Tables S3–S4 and Additional file 2: Fig. S6–S7). Compound **4** was the only predicted cWP derivative detected in LC–MS/MS analysis of samples obtained upon co-expression of CDPS-*S*sp5123 and CDO-*S*sp5123 (Fig. 3f). NMR characterization showed that **4** contains a Cα-Cβ double bond on the prolyl moiety of cWP (Additional file 1: Tables S3–S4 and Additional file 2: Fig. S8–S9). Co-expression of CDPS-*Said*5739 and CDO-*Said*5739 resulted in the production of **5** as the unique CDP derivative (Fig. 3h) which was shown by NMR to correspond to the *Z* isomer of cW∆L (Additional file 1: Tables S3–S4 and Additional file 2: Fig. S10–S11).

### In vivo evaluation of CDOs for CDP dehydrogenation in combinatorial engineering experiments

**Fig. 4:**
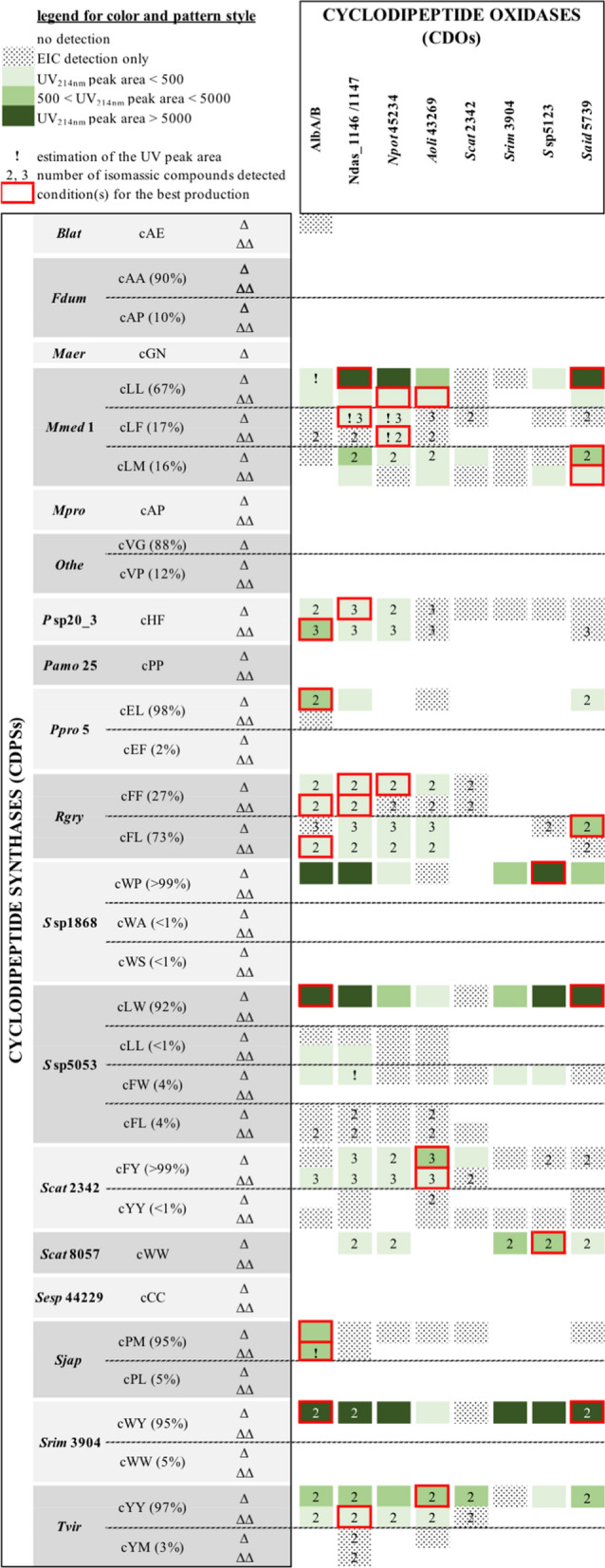
Efficiency of CDOs for in vivo dehydrogenation of CDPs. One CDPS and one CDO were co-expressed in *E. coli* and dehydrogenated CDPs were detected in culture supernatants by LC-MS/MS. CDPSs are indicated on the left by the suffix used to design them in Additional file 1: Table S2. The CDPs they synthesize are also indicated with percentages representing the proportion of each CDP (ratio of UV peak areas) as observed in an experiment without CDO expression (Additional file 1: Table S9). Δ and ΔΔ designate compounds with a [M + H]^+^ parent ion with a *m*/*z* value corresponding to di-dehydrogenated and tetra-dehydrogenated CDP, respectively. Co-expressed CDOs are indicated at the top on the right. The level of detection of each compound is indicated according to data in Additional file 1: Table S5 by a colour and pattern style (top left). When several compounds were detected for one *m*/*z* value, their number is indicated, and data reported in this figure correspond to those of the most highly produced compound. The best conditions for producing derivatives of the major CDPs produced by CDPSs are indicated by red rectangles

Combinatorial engineering of natural product biosynthetic pathways consists of co-expressing enzymes from different pathways in a suitable host to create unnatural biosynthetic pathways and achieve greater chemical diversity. We evaluated CDOs using such an approach by co-expressing them with CDPSs known to synthesize different CDPs. Eighteen CDPSs were selected for the variety of CDPs they synthesize (Additional file 1: Table S2). We co-expressed each of them with each of eight selected CDOs (AlbA/B, Ndas_1146/1147, CDO-*Npot*45234, CDO1-*Aoli*43269, CDO-*Scat*2342, CDO-*Srim*3904, CDO-*S*sp5123, and CDO-*Said*5739) in *E. coli* and evaluated the production of CDPs and their dehydrogenated derivatives in culture supernatants by LC–MS/MS. CDPs detected in the CDPS+/CDO– control experiments (Additional file 1: Table S5) were in accordance with previously published CDPS characterizations [19, 23], except for CDPS-*Mmed*1, which was found to synthesize cLM, in addition to cLL and cLF. Data for the detection of the dehydrogenated CDPs are reported in Additional file 1: Table S5 and summarized in Fig. 4. Two groups could be identified in terms of substrate acceptance in vivo, corresponding to broad-spectrum (AlbA/B, Ndas_1146/1147, CDO-*Npot*45234, and CDO1-*Aoli*43269) and narrow-spectrum CDOs (CDO-*Scat*2342, CDO-*Srim*3904, CDO-*S*sp5123, and CDO-*Said*5739). In addition to accepting a larger spectrum of substrates, broad-spectrum CDOs produced tetra-dehydrogenated CDPs more frequently and in greater amounts than narrow-spectrum CDOs. In terms of the nature of the converted CDPs, CDOs prefer CDPs with at least one hydrophobic side chain and conversion was never or poorly detected for cAE, cAA, cGN, cAP, cGV, cPP, cCC, cWA, and cWS. Except for cWA and cWS, these CDPs are comprised of amino acids carrying small side chains. We also observed different rates of conversion of CDPs, whether they were produced in high amounts or not (Additional file 1: Table S5 and Fig. 4). For example, cWW produced by CDPS-*Scat*8057 (UV_214_ peak area = 18,590) was converted upon co-expression of five different CDOs, whereas its conversion was not detected when it was produced by CDPS-*Srim*3904 (UV_214_ peak area = 306). The same was true for cLL, cFL, and cYY. Finally, the number of dehydrogenated derivatives detected for a given *m*/*z* value ranged from one to three, as previously observed for characterized CDOs [14, 18, 30], and were initially differentiated by their retention time.

One interesting point concerns the utility of these CDOs to produce a desired derivative in large quantities. The best combination(s) to produce the derivatives of the major CDPs produced by CDPSs are highlighted in Fig. 4 (red rectangles). Six of the eight tested CDOs were used in these combinations, two of them belonging to the so-called narrow-spectrum group. Notably, conversion of cWW into ∆cWW was best upon co-expression of CDO-*S*sp5123, whereas the associated CDPS-*S*sp5123 did not synthesize cWW. In most cases, several CDOs may be used for the conversion of one type of CDP. Certain CDPs (cLL, cWL, cWP, cWY, and cYY) are even very good substrates for several CDOs. Conversely, the production of significant amounts of dehydrogenated cPM was observed only upon co-expression of AlbA/B. Our combinatorial biosynthesis results provide the basis for the choice of CDOs to be used for the bioproduction of 2,5-DKPs in *E. coli*.

## Discussion

CDOs catalyse the dehydrogenation of the Cα-Cβ bond of CDPs in 2,5-DKP natural product biosynthetic pathways. Although the number of CDO-containing pathways identified in genomes has exploded in the last years, the number of studied CDOs has remained limited. We characterized the activity of seven previously undescribed CDOs to expand our knowledge of these pathways and evaluated the usefulness of CDOs for CDP modification in combinatorial engineering experiments to increase the toolkit of enzymes for 2,5-DKP chemical diversification.

We assessed the activities of the seven original CDOs by co-expressing each in *E. coli* with the associated CDPS in the BGC and characterizing the products by LC–MS/MS, LC-HRMS, and NMR spectroscopy. Six of the seven studied CDOs efficiently catalysed CDP dehydrogenation. Mass spectrometry and NMR data clearly support the production of cW∆Y by CDO-*Srim*3904, cW∆P by CDO-*S*sp5123, cW∆L by CDO-*Said*5739, and cY∆F and c∆YF by CDO1-*Aoli*43269 (Fig. 3, Table 1, Additional file 2: Fig. S2–S11). LC–MS/MS and LC-HRMS data showed that CDO-*Scat*2342 produced cY∆F and c∆YF, as the retention times and MS^2^ spectra of the two compounds were very similar to those of cY∆F and c∆YF produced by CDO1-*Aoli*43269 (Fig. 3, Table 1, Additional file 2: Fig. S1). Concerning CDO-*Npot*45234, the dehydrogenation of several cFX cyclodipeptides is supported by LC-HRMS data, cF∆F being produced in larger amounts based on the UV and EIC peak areas measured in LC–MS/MS and LC-HRMS chromatograms, respectively (Fig. 3 and Table 1). Finally, we did not observe a significant conversion of cWW by CDO2-*Aoli*43269, as di- and tetra-dehydrogenated cWW were only detected in trace amounts by LC-HRMS. We cannot exclude that CDO2-*Aoli*43269 may have little activity under our experimental conditions, thus resulting in the extremely low conversion of cWW. However, inspection of the genomic environment of CDPS2-*Aoli*43269 shows the presence of a cytochrome P450 (P450) gene (Additional file 2: Fig. S12). Many P450s have been described in 2,5-DKP biosynthetic pathways, and this encoded P450 may be involved in the modification of cWW before its conversion by CDO.

Our data on the active CDOs provide details on several biosynthetic pathways in which they are involved (Additional file 2: Fig. S12). CDO-*Srim*3904 is probably part of a guanitrypmycin biosynthetic pathway (Fig. 1). Its activity is similar to that of Gut(BC)_24309_, and the P450 and methyl transferase encoded by genes surrounding the CDO-*Srim*3904 gene share high sequence identity with the P450 GutD_24309_ and the methyl transferase GutE_24309_ involved in guanitrypmycin biosynthesis (83% over 80% of the entire sequence of GutD_24309_ and 79% over 86% of the entire sequence of GutE_24309_, respectively) [11]. The genes of CDO-*Said*5739 and CDO-*S*sp5123 are colocalized with three genes encoding a terpene cyclase (TC), a CDPS, and a phytoene synthase-like prenyl transferase (PT). A similar organization, but lacking the CDO genes, is also observed in *Streptomyces youssoufiensis* OUC6819. Recently, the TC/CDPS/PT proteins were shown to direct the synthesis of diketopiperazine-terpenes, called pre-drimentines and drimentines [29]. In these pathways, the tryptophanyl-containing CDP cWX synthesized by the CDPS is converted to pre-dimentine by the PT, which adds a farnesyl moiety to the indole ring. Then, the TC catalyses cyclisation of the farnesyl group, resulting in drimentines. A diversity of drimentines was observed, resulting from the various CDPs synthesized by each CDPS, i.e. cWL by CDPS-*Said*5739, cWP by CDPS-*S*sp5123, and cWX by the CDPS of *S. youssoufiensis* [29]. Our results showing the activity of CDO-*Said*5739 and CDO-*S*sp5123 on cWL and cWP, respectively, suggest that more complex drimentines can be obtained. Furthermore, the presence of methyl transferase genes in the vicinity of the TC/CDPS/PT genes could also account for the chemical diversification of drimentines. Insights into the corresponding biosynthetic pathways of the CDPS/CDO pairs from *A. oligospora* (pathway 1) and *S. catenulae* are more limited. The activities of these two pairs are similar, with the production of di- and tetra-dehydrogenated derivatives of cYF. Examination of the genomic environment allows identification of genes encoding potential 2,5-DKP tailoring enzymes that are clearly different between the two organisms (Additional file 2: Fig. S12). However, in the absence of other data, it is difficult to predict the fate of these compounds in the biosynthetic pathways, except that the end products are probably different.

We investigated the utility of eight CDOs for in vivo CDP diversification in combinatorial experiments with 18 CDPSs. We detected significant conversion of 14 of the 27 different CDPs synthesized by CDPSs in vivo into dehydrogenated derivatives (Fig. 4). Globally, CDOs show a preference for CDPs bearing large hydrophobic side chains, such as Leu, Phe, Tyr, or Trp. CDPs carrying small or polar side chains are poor substrates, and cLL, cFL, cFF, cFY, cLW, cWY, and cYY are relatively good substrates for CDOs. We defined broad- and narrow-spectrum CDOs according to the range of converted CDPs. These differences in in vivo activity may arise from the catalytic properties specific to each CDO, as well as from other uncontrolled factors in our experiments, such as the amount of active CDO produced in bacteria or the bioavailability of the CDPs. Furthermore, this narrow-/broad-spectrum distinction does not predetermine the usefulness of a particular CDO. For example, we observed the best conversion rates for cLM (*Mmed*1), cWW (*Scat*8057), and cWP (*S*sp1868) upon co-expression of narrow-spectrum CDOs. Certain CDPs were efficiently converted by a restricted number of CDOs, such as cPM (*Sjap*) and cEL (*Ppro*5), which are good substrates for only AlbA/B, or cWW, for which the conversion to the dehydrogenated derivative was best observed with two narrow-spectrum CDOs. Except for CDO-*Scat*2342, of which its co-expression did not induce a large range of dehydrogenated derivatives, all other tested CDOs may be useful in generating novel dehydrogenated compounds, thus highlighting the interest of evaluating diverse enzymes for desired biotransformation.

We observed an effect of the level of production of a CDP on its possible conversion by a CDO. Indeed, several highly-produced CDPs gave rise to dehydrogenated derivatives with several CDOs (cLL produced by *Mmed*1, cFL produced by *Rgry*, cWW produced by *Scat*8057), whereas the same compounds produced by other CDPSs, but at a lower level (cLL produced by *S*sp5053, cFL produced by *S*sp5053, cWW produced by *Srim*3904), were not transformed by any CDO or only poorly transformed (Fig. 4 and Additional file 1: Table S5). Furthermore, unconverted CDPs were observed in most experiments with co-expression of a CDO (Additional file 1: Table S5), indicating that higher levels of dehydrogenated derivatives could be potentially produced. The CDPS/CDO couple must be chosen carefully, depending on the desired transformation, but synthetic biology approaches aimed at reducing substrate escape by gene fusion or enzyme scaffolding could be developed to optimize the biosynthetic pathway from amino acyl-tRNAs to dehydrogenated CDPs [31].

Recently, S. M. Li et al. assessed the activity of three CDOs, AlbA/B, Ndas_1146/1147, and CDO-Np from *Nocardiopsis prasina* (CDO-*N*spL17 in Fig. 2 and Additional file 1: Table S1) on chemically synthesized CDPs in either in vitro assays using CDO-containing cell-free extracts or in feeding experiments to *E. coli* bacteria expressing each CDO [14]. The authors mentioned the difficulty of the three CDOs to generate ∆Trp-containing derivatives [14]. In spite of the larger number of CDOs tested in this study and their distribution on the phylogenetic tree, we observed similar results, with few ∆Trp-containing derivatives. Indeed, the various ∆cWL, ∆cWY, and ∆cWP produced by several CDPS/CDO combinations had similar LC–MS/MS characteristics (retention time and MS^2^ fragmentation pattern), indicating that they correspond to the compound identified by NMR spectroscopy as the *Z*-isomer of the cW∆X derivative (Fig. 4, Additional file 1: Table S5). ∆Trp-containing derivatives were observed only upon cWW conversion, the most efficient conditions being with CDO-*S*sp5123 co-expression. Intriguingly, CDO-*S*sp5123 can produce ∆Trp derivatives of cWW, but very few or no ∆Trp derivatives of cWL, cWY, or cWP. Our full biosynthetic strategy and the mutasynthetic approaches developed by Li et al. provide two different options for the derivatization of CDPs, each offering advantages and drawbacks. A major benefit of the mutasynthetic approaches resides in the large panel of testable CDPs, provided that they are chemically synthesizable. However, we recently described the in vivo incorporation of unnatural amino acids into CDPs by CDPSs and the production of prenylated CDPs by reprogrammed *E. coli* bacteria, thus enlarging the panel of biosynthesized 2,5-DKPs [32, 33]. Our study lays the foundations to evaluate the in vivo activity of CDOs in such more complex unnatural 2,5-DKPs biosynthetic pathways.

## Methods

### Nomenclature

We homogenized the nomenclature of the CDPS and CDO proteins for clarity. We denoted the two CDO subunits CDOA and CDOB. Furthermore, we added a suffix to indicate the native producing organism. For example, CDPS-*Srim*3904 and CDO-*Srim*3904 denote the CDPS and CDO originating from *Streptomyces rimosus* strain NRRL WC-3904. The two CDO subunits are denoted CDOA-*Srim*3904 and CDOB-*Srim*3904. We used the terms CDPS1/CDO1 and CDPS2/CDO2 to distinguish between the enzymes of two CDPS- and CDO-containing pathways found in the same organism. The names of the CDPS and CDO belonging to previously studied BGCs are conserved, i.e. AlbC and AlbA/B of *Streptomyces noursei* ATCC 11455 [9], Ndas_1145 and Ndas_1146/1147 of *Nocardiopsis dassonvillei* DSM 43111 [10], GutA_24309_ and Gut(BC)_24309_ of *Streptomyces monomycini* NRRL B-24309 [11], and CDPS-Np and CDO-Np of *Nocardiopsis prasina* [13, 14].

Throughout the text, cXX refers to CDPs constituted by l-amino acids, X representing each amino acid in the one-letter code. The dehydrogenation of the Cα-Cβ bond is indicated by the symbol ∆. For example, c∆YW refers to *cyclo*(dehydrotyrosyl-l-tryptophanyl).

### Bioinformatics

*Protein Sequence Similarity Network.* We used facilities at the Enzyme Function Initiative-Enzyme Similarity Tool (EFI-EST) website to create a protein sequence similarity network (SSN) [28]. First, a set of 1610 protein sequences was retrieved from the Uniprot database using CDOA-*Snou*11455 (AlbA) as the entry and an E-value of 5. Then the SSN was generated using an E-value of 10^−30^. Finally, the SSN was visualized and edited using Cytoscape version 3.5.1. In this SSN, two nodes (each representing one protein) are linked by an edge if the two protein sequences have more than 26.5% sequence identity.

*Phylogenetic Tree calculation.* We searched the NCBI protein database for CDOA and CDOB sequences using CDOA-*Snou*11455 (AlbA) as the entry (June 2017). We selected CDOA sequences for which the CDOA gene is in proximity of a CDPS gene and a CDOB-encoding gene. We thus constituted two sets of 32 CDOA and CDOB subunits (Additional file 1: Table S1). The CDOA and CDOB phylogenetic trees were calculated using bioinformatic tools at EMBL-EBI (https://www.ebi.ac.uk/). Two multiple sequence alignments of the CDOA and CDOB sequences were generated using Clustal Omega set with default settings. Alignments were then sent to Simple Phylogeny for tree calculation using the UPGMA clustering method (default setting parameters). Finally, trees were edited using iTol (https://itol.embl.de/).

### DNA manipulation and plasmids

DNA was manipulated according to standard procedures [34]. Molecular biology enzymes were purchased from New England Biolabs. Bacteria of *E. coli* strain DH5α were used for cloning procedures and plasmid propagation (Invitrogen). They were grown in LB medium at 37 °C unless otherwise stated. When needed, ampicillin and kanamycin were used at 200 µg/ml and 30 µg/ml, respectively. Competent cells were prepared and transformed according to a high-efficiency transformation protocol [35]. Plasmids were purified using the GenElute™ Plasmid MiniPrep kit or the GenElute™ HP Plasmid MidiPrep kit (Sigma-Aldrich). DNA fragments were purified from agarose gels using NucleoSpin Gel and PCR Clean-up (Macherey–Nagel). For DNA sequencing, plasmids were dialyzed against water using 0.025 µm VSWP membrane filters (Millipore) and sent to Eurofins Genomics in Mix2Seq kit tubes.

CDPSs were expressed from pIJ196 derivatives (ColE1 replicon, ampicillin resistance) under the control of the PT5-*lacO* promoter [23]. Most of the pIJ196-CDPS plasmids used in this study have been described previously and the characteristics of the encoded CDPSs are given in Additional file 1: Table S2 [19, 23]. We constructed pIJ196-CDPS1-*Aoli*43269 and pIJ196-CDPS-*Said*5739. Briefly, the synthetic genes were obtained from Life Technologies SAS according to the sequences described in Additional file 1: Table S6. They were cloned in pIJ196 as previously described and the final constructs were sequenced to verify the promoter region and the cloned gene [23].

CDOs were expressed from pIJ194 derivatives (RSF replicon, kanamycin resistance) under the control of the PT7-*lacO* promoter [33]. CDOA and CDOB genes overlap in genomes, and previous studies have shown that the expression of CDOA and CDOB from different cistrons is detrimental to CDO production [9, 10]. Thus, the CDO genes used in this study corresponded to the native genes with introduction of a *Nco*I restriction site 5′ and a *Xho*I restriction sites 3′ for cloning and elimination of undesired restriction sites (Additional file 1: Table S7). The gene of CDO-*Snou*11455 (AlbA/B) was obtained by PCR SOEing to eliminate a *Nco*I site in the CDOA gene. Briefly, PCR1 and PCR2 were performed using Phusion DNA polymerase, plasmid pSL150 as matrix, and oligonucleotides and the PCR conditions described in Additional file 1: Table S8 [9]. After purification from an agarose gel, the products of PCR1 and PCR2 were used as matrices in PCR3 to obtain the final DNA fragment (Additional file 1: Table S8). After purification from an agarose gel, it was digested with *Nco*I and *Xho*I and the digested DNA fragment cloned between the *Nco*I and *Xho*I sites of pIJ194. The sequence of the promoter region and cloned DNA of pIJ194-CDO-*Snou*11455 was verified. Synthetic genes encoding the other CDOs studied herein were obtained from Life Technologies SAS, according to Additional file 1: Table S7, and were provided cloned in a supplier vector. After digestion with *Nco*I and *Xho*I, we purified the CDO gene-containing DNA fragment from an agarose gel and cloned it between the *Nco*I and *Xho*I restriction sites of pIJ194. Sequences of the pIJ194-CDO plasmids were verified.

### Expression of CDPS and CDO genes in *E. coli* bacteria

Expression of CDPS and CDO genes for 2,5-DKP production was performed in *E. coli* bacteria of strain BL21AI (Invitrogen). Competent cells of BL21AI were prepared using the frozen storage III procedure described by Hanahan [36]. We used a two-transformation procedure to introduce pIJ194 derivatives and pIJ196 derivatives into BL21AI. First, plasmid pIJ194 and its pIJ194-CDO derivatives were used to transform BL21AI bacteria using 30 µg/ml kanamycin. Second, competent cells of transformants were prepared according to Hanahan after growing in LB plus kanamycin and used for transformation with pIJ196 and its pIJ196-CDPS derivatives. Double transformants were selected at 37 °C on LB plates containing 30 µg/ml kanamycin and 200 µg/ml ampicillin. They were used to inoculate M9 minimal medium containing 0.5% glucose plus the corresponding antibiotics, trace elements, and vitamins [23]. After 20 h growth at 37 °C, we used the starter cultures to inoculate prewarmed M9 minimal autoinducing medium, containing 0.05% glucose, 0.5% glycerol, 0.2% lactose, and 0.02% arabinose plus trace elements and vitamins, at a 1:100 volume ratio [37]. After 3 h growth at 37 °C in a rotary shaker (210 rpm), production cultures were transferred to 20 °C in a rotary shaker for 44 h. For co-expression of the CDPS and CDO genes from the same biosynthetic pathway, we used 5-ml starter cultures in 50 ml Falcon tubes and 25-ml production cultures in 250 ml Erlenmeyer flasks. For combinatorial engineering experiments with co-expression of CDPS and CDO genes from different pathways, starter cultures and production cultures were made in 24 well-plates (deep well, round bottom; Dutscher) containing 2 ml medium per well and covered with a sterile porous membrane (VWR).

For large-scale production of 2,5-DKP, we performed 350-ml cultures in 3 L Erlenmeyer. The media composition, inoculation ratio, and growth conditions were preserved, except for transfer to 20 °C that occurred after 3 h growth at 37 °C.

The produced 2,5-DKPs were detected in and recovered from culture supernatants as described below.

### LC–MS/MS analysis

CDPs and dehydrogenated CDPs were found in the culture supernatants upon expression of CDPS and CDO in *E. coli* bacteria. Production cultures were acidified to 2% formic acid to stop growth and the culture supernatants were recovered by centrifugation. For the co-expression of CDPS and CDO genes from the same biosynthetic pathway, 5 ml acidified supernatant was treated by solid phase extraction (SPE) using Strata™-X polymeric sorbent following the manufacturer’s instructions (30 mg per tube, Phenomenex). After washing with 5% methanol, elution was performed with 500 µl methanol and the samples conserved at 4 °C in Eppendorf tubes sealed with parafilm until LC–MS/MS analysis. For the co-expression of CDPS and CDO genes from different biosynthetic pathways for combinatorial engineering experiments, acidified supernatants were analysed by LC–MS/MS.

LC–MS/MS analyses were performed on an Elute HPLC (Bruker Daltonics) coupled via a split system to an AmaZon SL ion-trap mass spectrometer set in positive mode (Bruker Daltonics). Samples were loaded onto an Excel 3 C18-PFP column (150 × 4.6 mm, 3 µm, 100 Å, ACE) equilibrated in 0.1% formic acid in water (solvent A) at 0.6 ml/min. After 5 min in solvent A, a 0–50% linear gradient of solvent B (0.1% formic acid in 90:10 CH_3_CN:H_2_O) in A was applied over 20 min at the same flow rate.

### HRMS analysis

SPE-treated supernatants were analysed by LC-HRMS. Two microliters of the eluates in methanol were injected as technical duplicates in an Ultimate 3000 (Dionex) and separated on a C18 column (3.5 μm, 1 mm × 150 mm, Zorbax-Microbore SB-C18, Agilent Technologies) at a flow rate of 50 μl/min with a 50 min linear gradient from 100% solvent A (2% CH_3_CN, 0.1% formic acid in H_2_O) to 80% solvent B (98% CH_3_CN, 0.1% formic acid in H_2_O). The LC was coupled to an LTQ Orbitrap XL (Thermo Fisher Scientific) operating in positive-ion mode. All MS spectra were acquired on the Orbitrap with the following parameters: *m*/*z* range, 100–1500; resolution, 100000; AGC target, 2 × 10^5^; maximum injection time, 400 ms; lockmass enabled (*m*/*z* 279.15909).

Raw files were opened with Thermo Xcalibur Qual Browser (v. 3.0.63) and manually interpreted. Observed *m*/*z* values were averaged over the chromatographic peak. Retention time (RT) was reported at the apex of the peak. Reported extracted ion chromatograms were used as is. All species considered in this study were systematically searched in every raw file.

### Purification of 2,5-DKPs

Large-scale cultures for the production of 2,5-DKPs were acidified with formic acid (final concentration 2%) and centrifuged to recover the supernatants. Acidified supernatants were loaded onto SPE columns (Strata™-X Giga, 1 g per tube, Phenomenex) conditioned according to the manufacturer’s instructions. A maximum of 500 ml of supernatant was applied per tube. After washing with 5% methanol, elution was performed with 10 ml methanol. Purification of the 2,5-DKPs was continued by semi-preparative HPLC (Hitachi, LP1100/LP3101) using a Purospher Star RP-18e column (250 × 10 mm, 5 µm, VWR) and two solvent conditions (solvent A: 0.1% formic acid; solvent B: 90:10 CH_3_CN:H_2_O in 0.1% formic acid) at a flow rate of 4.75 ml/min. Details of the chromatographic conditions optimized for each purified compound are given in Additional file 1: Table S9. Identification of the fraction containing the desired compound, pooling of fractions, lyophilization, and the evaluation of purity were performed as previously described [33].

### NMR experiments

NMR experiments were recorded on a Bruker Avance III spectrometer equipped with a TCI cryoprobe and operating at a ^1^H frequency of 500.3 MHz. Spectra were recorded at 26 °C in DMSO-*d*_6_ (Eurisotop). ^1^H, ^13^C, and ^15^N resonances were assigned through the analysis of 1D ^1^H, 1D ^13^C DEPTQ, 2D ^1^H-^1^H COSY, 2D ^1^H-^1^H ROESY, 2D ^1^H-^13^C HSQC, 2D ^1^H-^13^C HMBC, and 2D ^1^H-^15^N HMBC. ^1^H and ^13^C chemical shifts were referenced to the DMSO solvent signal (*δ* 2.50 and 39.5 ppm, respectively) and ^15^N chemical shifts were referenced indirectly. NMR experiments were processed and analysed using the Bruker Topsin 3.5 program. NMR spectra and NMR data for cY∆F, c∆YF, cW∆Y, cW∆P, and cW∆L are presented in Additional file 2: Fig. S2–S11 and Additional file 1: Table S3. Heteronuclear ^3^*J*_Hβ-CO_ coupling constants (Additional file 1: Table S4) were measured on a 2D ^1^H-^13^C HSQMBC IPAP experiment [38].

## Conclusions

*E. coli* is a widely used genetic background for the production of a large range of natural products. Here, we added the production of various dehydrogenated 2,5-DKPs from carbon sources to the *E. coli* catalogue. We used the concomitant recombinant expression of CDPSs and CDOs, which allowed the recovery of dehydrogenated 2,5-DKPs directly in the culture supernatants. After the characterization of the activities of six novel CDOs, we implemented a combinatorial engineering approach based on two sets of enzymes consisting of 18 CDPSs and 8 CDOs. Among the 144 combinations, we identified the best pairs for the production of the highest levels of many dehydrogenated 2,5-DKPs. This work constitutes the first step toward the bioproduction of more complex 2,5-DKPs.

## Supplementary information

**Additional file 1: Table S1.** Database information relative to CDOA and CDOB subunits. **Table S2.** Characteristics of the CDPSs encoded in the pIJ196-CDPS plasmids used in this study. **Table S3.** NMR data for cYΔF, cΔYF, cWΔY, cWΔP, and cWΔL. **Table S4.** ^3^J_Hβ-CO_ coupling constants and key ROEs observed for the dehydroaminoacid. **Table S5.** Detection of CDPs and their derivatives in the supernatants of cultures of bacteria expressing CDPSs alone or in combination with diverse CDOs. **Table S6.** Database information and sequence data relative to previously uncharacterized CDPSs. **Table S7.** Sequences of the genes encoding CDOs. **Table S8.** Oligonucleotides and PCR conditions for the construction of pIJ194-CDO-*Snou*11455. **Table S9.** HPLC conditions for the purification of 2,5-DKPs.

**Additional file 2: Fig. S1**. Mass spectra of predicted ΔcFY (*m*/*z* 309). **Fig. S2**. 1D ^1^H NMR spectrum (with presaturation of residual water) and 1D ^13^C NMR DEPTQ spectrum of cYΔF in DMSO-*d*_6_. **Fig. S3**. 2D ^13^C-^1^H HSQC spectrum and 2D ^13^C-^1^H HMBC spectrum of cYΔF in DMSO-*d*_6_. **Fig. S4**. 1D ^1^H NMR spectrum (with presaturation of residual water) and 1D ^13^C NMR DEPTQ spectrum of cΔYF in DMSO-*d*_6_. **Fig. S5**. 2D ^13^C-^1^H HSQC spectrum and 2D ^13^C-^1^H HMBC spectrum of cΔYF in DMSO-*d*_6_. **Fig. S6**. 1D ^1^H NMR spectrum and 1D ^13^C NMR DEPTQ spectrum of cWΔY in DMSO-*d*_6_. **Fig. S7**. 2D ^13^C-^1^H HSQC spectrum and 2D ^13^C-^1^H HMBC spectrum of cWΔY in DMSO-*d*_6_. **Fig. S8**. 1D ^1^H NMR spectrum and 1D ^13^C NMR DEPTQ spectrum of cWΔP in DMSO-*d*_6_. **Fig. S9**. 2D ^13^C-^1^H HSQC spectrum and 2D ^13^C-^1^H HMBC spectrum of cWΔP in DMSO-*d*_6_. **Fig. S10**. 1D ^1^H NMR spectrum and 1D ^13^C NMR DEPTQ spectrum of cWΔL in DMSO-*d*_6_. **Fig. S11**. 2D ^13^C-^1^H HSQC spectrum and 2D ^13^C-^1^H HMBC spectrum of cWΔL in DMSO-*d*_6_. **Fig. S12**. Genomic environment of the CDO genes and predicted biosynthetic pathways.

## Data Availability

The datasets used and/or analysed during the current study are available from the corresponding author on reasonable request.

## Abbreviations

2,5-DKP: 2,5-Diketopiperazine
Aminoacyl-tRNA: aa-tRNA
BGC: Biosynthetic gene cluster
CDO: Cyclodipeptide oxidase
CDOA: CDO subunit A
CDOB: CDO subunit B
CDP: Cyclic dipeptide
CDPS: Cyclodipeptide synthase
RT: Retention time
SPE: Solid phase extraction
SSN: Sequence similarity network
