# Supplementary material for: In vivo characterization of the activities of novel cyclodipeptide oxidases: new tools for increasing chemical diversity of bioproduced 2,5-diketopiperazines in Escherichia coli
Source: Microb Cell Fact. 2020 Sep 7;19:178. doi: 10.1186/s12934-020-01432-y (PMC7487605; doi:10.1186/s12934-020-01432-y)
Supplement: Supplementary file 1 — Additional file 1: Table S1. Database information relative to CDOA and CDOB subunits. Table S2. Characteristics of the CDPSs encoded in the pIJ196-CDPS plasmids used in this study. Table S3. NMR data for cYΔF, cΔYF, cWΔY, cWΔP, and cWΔL. Table S4. 3JHβ-CO coupling constants and key ROEs observed for the dehydroaminoacid. Table S5. Detection of CDPs and their derivatives in the supernatants of cultures of bacteria expressing CDPSs alone or in combination with diverse CDOs. Table S6. Database information and sequence data relative to previously uncharacterized CDPSs. Table S7. Sequences of the genes encoding CDOs. Table S8. Oligonucleotides and PCR conditions for the construction of pIJ194-CDO-Snou11455. Table S9. HPLC conditions for the purification of 2,5-DKPs. [file 12934_2020_1432_MOESM1_ESM.docx]

**Additional File 1**

***In vivo* characterization of the activities of novel cyclodipeptide oxidases: new tools for increasing chemical diversity of bioproduced 2,5-diketopiperazines in *Escherichia coli***

Fabien Le Chevalier, Isabelle Correia, Lucrèce Matheron, Morgan Babin, Mireille Moutiez,

Nicolas Canu, Muriel Gondry, Olivier Lequin, and Pascal Belin

**Table S1**: Database information relative to CDOA and CDOB subunits.

**Table S2**: Characteristics of the CDPSs encoded in the pIJ196-CDPS plasmids used in this study.

**Table S3**: NMR data for cYΔF, cΔYF, cWΔY, cWΔP, and cWΔL.

**Table S4**: ^3^J_Hβ-CO_ coupling constants and key ROEs observed for the dehydroaminoacid.

**Table S5**: Detection of CDPs and their derivatives in the supernatants of cultures of bacteria expressing CDPSs alone or in combination with diverse CDOs.

**Table S6**: Database information and sequence data relative to previously uncharacterized CDPSs.

**Table S7**: Sequences of the genes encoding CDOs.

**Table S8**: Oligonucleotides and PCR conditions for the construction of pIJ194-CDO-*Snou*11455.

**Table S9**: HPLC conditions for the purification of 2,5-DKPs.

**Table S1.** Database information relative to CDOA and CDOB subunits.

| **CDO name** | **Organism** |  | **CDOA and CDOB sequences (FASTA format)** | | |
| --- | --- | --- | --- | --- | --- |
|  |  |  | **CDOA** |  | **CDOB** |
| CDO2-*Aoli*43269 | *Actinomadura oligospora* ATCC 43269 |  | >WP_084338280.1 hypothetical protein [Actinomadura oligospora] MTTDTAPPAPAAPQATSAPPAPPPAEHAVDPAGVLQVIRTRAVVREFTGEPVSDDDAKAL  AAALVAAPNGANLQAWAFVIVRRPQLLLSVRAFAPGVFAIPALILVACLDHSRAADESDT  RTHKEGRLCVAMAVENFLLAAHALGLGACPSSSFLPGPIRVLLDLPDHLEPVMVVSAGHP  AHPPKPAPRRDIDEVVHYDNYA |  | >WP_051468277.1 hypothetical protein [Actinomadura oligospora]  MTTTPESTRREAGLSRRESELAQLAAYLLASSRSLLDDP  AVYGSFRLIDAARRTLLILESEGVANADFTAVRTQIEEV  VRAKTEVDVQAFLDTLCLQMAHALKAADPDTLDTNL |
| AlbA/B | *Streptomyces noursei* ATCC11455 |  | >AAN07907.1 AlbA [Streptomyces noursei ATCC 11455]  MRRHPSHSPYRGGCEVRPKRRGLMLAHSSSESPPESLPDAWTVLKTRTAVRNYAKEPVD  DALIEQLLEAMLAAPTASNRQAWSFMVVRRPAAVRRLRAFSPGVLGTPAFFVVACVDRS  LTDNLSPKLSQKIYDTSKLCVAMAVENLLLAAHAAGLGGCPVGSFRSDIVTSMLGIPEHIE  PMLVVPIGRPATALVPSQRRAKNEVVNYESWGNRAAAPTA |  | >AAN07908.1 AlbB [Streptomyces noursei ATCC 11455]  MNPGETVLPPQLREEIALLAVYLLSSGRGLLEEPADYGI  YRCTDGARRALQLLDEHGGSTARLTAVRERLDEVMFA  PMGEDRDMGAILDDLCRQMADALPEIETP |
| CDO-*Salb*11814 | *Streptomyces albulus* CCRC11814 |  | >WP_037634410.1 nitroreductase family protein [Streptomyces albulus] MLTQSSSESPPESLADALTVLKTRTAVRNYSKEPVDDALIERLLEAMLAAPTASNRQAWS  FVVVRRPAGVRQLRAFSPGVLGTPAFFVVACVDRSLTDNLSPKLSQTIYDTSKLCVAMAV  ENLLLAAHAFGLGGCPVGSFRSEIVKSMLGIPEHIEPMLVVPIGRPATALVPSPRRATNEVV  NYESWGNRAAAPTA |  | >WP_051273455.1 MULTISPECIES: hypothetical protein [Streptomyces]  MREDIALLAAFLLSSGRGLLDEPADYGIYRCTDGARR  VLQLLDEHGGSTARLTAVRERLDEVMFAPMGEDRD  MGEILDDLCRQMAGALPEIETP |
| CDO-*Salb*PD1 | *Streptomyces albulus* PD-1 |  | >EXU91236.1 nitroreductase [Streptomyces albulus PD-1]  MLTQSSSESPPESLADALTVLKTRTAVRNYSKEPVDDALIERLLEAMLAAPTASNRQAWS  FVVVRRPAGVRQLRAFSPGVLGTPAFFVVACVDRSLTDNLSPKLSQTIYDTSKLCVAMAV  ENLLLAAHAFGLGGCPVGSFRSEIVKSMLGIPEHIEPMLVVPIGRPATALVPSPRRATNEVV  NYESWGNRAAAPTA |  | >EXU91237.1 hypothetical protein [Streptomyces albulus PD-1]  MNPGETVLPPQLREDIALLAAFLLSSGRGLLDEPADYG  IYRCTDGARRVLQLLDEHGGSTARLTAVRERLDEVMF  APMGEDRDMGEILDDLCRQMAGALPEIETP |
| CDO-*Salb*660 | *Streptomyces albulus* NK660 |  | >AIA08474.1 nitroreductase [Streptomyces albulus]  MLTQSSSESPPESLADALTVLKTRTAVRNYSKEPVDDALIERLLEAMLAAPTASNRQAWS  FVVVRRPAGVRQLRAFSPGVLGTPAFFVVACVDRSLTDNLSPKLSQTIYDTSKLCVAMA  VENLLLAAHAFGLGGCPVGSFRSEIVKSMLGIPEHIEPMLVVPIGRPATALVSSPRRATNE  VVNYESWGNRAAAPTA |  | >AIA08473.1 hypothetical protein [Streptomyces albulus]  MNPGETVLPPQLREDIALLAAFLLSSGRGLLDEPADYG  IYRCTDGARRVLQLLDEHGGSTARLTAVRERLDEVMF  APMGEDRDMGEILDDLCRQMAGALPEIETP |
| CDO1-*Smon*24309 | *Streptomyces monomycini* strain NRRL B-24309 |  | >WP_030022688.1 nitroreductase family protein [Streptomyces monomycini]  MSKNPQKDSPPGELPDALHVLRTRSVVRNYAPEPVDASVIDQLLDAMLAAPSASNKQA  WSFVVVRDPASVRRLRAFSPGIIGTPAFCVLACVDQSLTKHHSPAAAENIHATSKLCVA  MAVENLLLAAHAVGLGGCPVHSFRKDAVGALLGLPEHIEPVLIVPIGRPASPPTPSERRD  KKEVVSHEVWGNHSATTPAV |  | >WP_030022686.1 hypothetical protein [Streptomyces monomycini]  MHEDVALLAAYLLSSARGLLAEPPEYGVYRCLDGARR  ALETLDQNGVSTPELSAVRRRLDDQMFSPMGGDELLP  GILDDVCMRLVDALKNMKSA |
| CDO-*Scel*2493 | *Streptomyces celluloflavus* strain NRRL B-2493 |  | >WP_110951963.1 nitroreductase family protein [Streptomyces celluloflavus]  MPKISSPEVPAETPTRTLPVTLPETLNVLRSRSVVRNYAPDRVDDSLIEQLLECMLAAPTA  SNKQAWSFVVVREPAGVRLLRAFSPGIIGTPAFVVVACLDRRLTDGLSGNISQKIYQTSK  LCVAMAVENLLLSAHALGLGGCPVSSFREGAVRLLLDLPTPIEPILMVPIGRPAQKLTPSE  RRDKNEVISYEIWGKDAATGSAA |  | >WP_110951962.1 hypothetical protein [Streptomyces celluloflavus]  MKSGEKTLPQDRLHEEIALLAAYLLSSGRGLLDEPAD  YGAYRCADAARRTLGILEQAGASTPQLSGIRHRLDEV  MFSPMGGDLHLPEILDDLCLQLAGALKELKTA |
| CDO-*Scaa*1322 | *Streptomyces caatingaensis* strain CMAA 1322 |  | >WP_078870981.1 nitroreductase family protein [Streptomyces caatingaensis]  MPKSPLPETQADVLNVIRTRTVLRNYAPETIDDALVDRMLECMLAAPTASNKQAWSFV  VVRDPANVRLLRAFSPGIIGTPSFVVVACVDRRLTAGLTGEISRKIYETSKLCVAMAVEN  LLLSAHALGFGGCPVSSFREEVLRHLLGLPEALEPVLLVPVGRAAQPPTPSDRRDKNEVI  SYEVWGNRTAEPAA |  | >WP_049714855.1 hypothetical protein [Streptomyces caatingaensis]  MKSGETVPQNRLHEDIALLAAYLLSSGRGLLNEPSD  YGAFRCADAARRTLEILERAGGSSPGLTEVRERLDG  VMFAPMGGDTDIAAVLDELCLKMADSLKNAASAPSS |

**Table S1.** Continued.

| **CDO name** | **Organism** |  | **CDOA and CDOB sequences (FASTA format)** | | |
| --- | --- | --- | --- | --- | --- |
|  |  |  | **CDOA** |  | **CDOB** |
| CDO-*Scat*2342 | *Streptomyces catenulae* strain NRRL B-2342 |  | >WP_078654370.1 nitroreductase family protein [Streptomyces catenulae]  MDVIRTRGVVRSYTDEPVGDELLDSLLEAMLAAPSASNKQAWAFLAVRAPERVR  RLRAFAPGIIGVPPLVLVACVDHARMTGDPHLRDVGSLCVAMAVENFLLAAHAQ  GLGGCPVSSFLAEPVQLLLGLPGHLEPLLLVPVGRPDQPLQPSPRRAPQEVVRHEY  WHPTHPG |  | >WP_051740007.1 hypothetical protein [Streptomyces catenulae]  MSTGTPRTPDDPGGEELALLAAYLLSSARRLLQEPPSYAL  YRLMDGARRVLALYADGGGDRPELVAVHAGLDDLLHQ  APNENRDYAALLDGMCRQMVAGLQLPAPEPAASAPS |
| CDO-*Said*5739 | *Streptomyces aidingensis* strain CGMCC 4.5739 |  | >SFD40970.1 Nitroreductase [Streptomyces aidingensis]  MEPTGEGEEVLRVLRGRAVVRRYTAEPVDDALLDRLLEVTVTAPTAANKQAWG  FLAVRAPRMVRCLRAFAPGMIGLPPLVVVACFDRDRAVGEPAAPWDAGMLCVA  MAVENLLLAAHAVGLGGCPVSSFDRAAVRRLVRLPPALEPLLLVPLGRPARIPEP  AARRERSEVIRYESWD |  | >SFD40938.1 hypothetical protein [Streptomyces aidingensis]  MSHGTEPPVPPVPPGPPASAAAEGLTLLAAFLLSSARG  LLNEPPGYGVARCADGARRTLELLDLCGGGPDPRLVR  VRERLEETMCGPMSAADFPALLDRALDEVVSVIEEGG  DREPGGLTGAG |
| Gut(BC)_24309_ | *Streptomyces monomycini* strain NRRL B-24309 |  | >WP_033038824.1 nitroreductase [Streptomyces monomycini]  MRRYTREPVDDALLDALLDVMPAAPTAANKQAWGFVAVRDPYTVRCLRAFAP  GMIGLPPLVVTACFDRDRAAREDSGPTDVGLLCVAMAVQNLLLAAHATGLGGC  PVSSFSRTAVGRLLALPPHLEPVLLVPVGHPADPVRPSARRHRDEVIRHGTWT |  | >WP_050502761.1 hypothetical protein [Streptomyces monomycini]  MAPGPDQPTTAPDLTDDIVLLAAFLLSSAHGLLDEPPA  YGPARCADGARRALELLDAYGEPDPALLRVRTQLEDA  MCGPMADVDLPSLLRTACEQMLDVITARRAGAPHVPAG |
| CDO-*Svar*3589 | *Streptomyces varsoviensis* strain NRRL B-3589 |  | >WP_048832786.1 nitroreductase [Streptomyces varsoviensis]  MRSRAVVRRYTDEPVDDALLAELLDVMPTAPTAANKQAWAFVAVRDPYAVRCL  RAFAPGMIGLPPLVVAACFDRDRAVREDSGPTDTGLLCLAMAVQNLLLAAHAAG  LGGCPVSSFSRTAAHRLLALPPHLEPVLLVPVGHPADPVRPSARRDRDEVIRHDVW  ISPARDSTGPDR |  | >WP_030877661.1 hypothetical protein [Streptomyces varsoviensis]  MTSGYLPPGTAPDLTDDIVLLAAYLLSSGHGLLDEPAD  YGPARCADGARRALELLDAYGGADPALLGVRRRLED  AMCGSMADVDLPTLLSTECEHILDVITARRASAPRPPA |
| CDO-*Srim*3904 | *Streptomyces rimosus* strain NRRL WC-3904 |  | >WP_050514429.1 nitroreductase [Streptomyces rimosus]  MLSQETAPEATGSATTAAAFLRLLRSRAVVRRYTDEPVADALLAELLDVMPTAPT  AANKQAWAFVAVRDPYAVRCLRAFAPGMIGLPPLVVAACFDRDRAVREDSGSQ  DTGLLCVAMAVQNLLLAAHASGLGGCPVSSFSRTAVHRLLALPHHLEPVLLVPV  GHPAAPVRPSARRDRDEVIHHDVWTSPTAASAGP |  | >WP_030659934.1 hypothetical protein [Streptomyces rimosus]  MTSGPPPPPRQPDLDDDIVLLAAFLLSSGHGLLDEPPAY  GPARCADGARRALELLDTHGTPDPALTRVREQLENAM  CGSMADVDLPSLLRTTCDQVLDVVMARRAGASRLL |
| CDO1-*Aoli*43269 | *Actinomadura oligospora* ATCC 43269 |  | >WP_026411673.1 nitroreductase [Actinomadura oligospora]  MAGRSTDEPQREQPEAVRVLLSRTAVRQFTDQPVGDDLVGPMSEALVAAPSASN  RQAWAFVLVRDRRVVRLVQAFSPGVLATPPLIVVACFDRSRGVGDTGERYDECL  LCVAMAVQNLLLAAHALGLGGCPVASFRERPLRRVLGLPAHIDPILLVSVGHPVR  LNPHPVRRDPSEVIHHDVWSGGDAPRAR |  | >WP_026411672.1 hypothetical protein [Actinomadura oligospora]  MTSGAAATPRELGEELLLLAAYLLSSGRGLFDEPPAYGP  LRCADAARRVLGLVERAGIEHPEIHALRAELDELFFGPM  GDGNIRELLDLLCERTGALLHESDVIQTSGE |
| CDO-*Ssp*E11A | *Streptomyces* sp. AmelKG-E11A |  | >SCK05932.1 Nitroreductase [Streptomyces sp. AmelKG-E11A]  MRVLRTRSAVRQFTDQPVSDGQLEQLIDAILAAPTGGNKQAWAFVAVRDPRTLR  LVRAFSPGMIEPPPLVVVACFDRSRAVKDDGEFWDEGLLCVAMAVQNLLLAAHC  LGLGGCPAASFRRGSVAAILGLPGHLEPLLLVPLGHPARDLVSPPRRDRSEVVSHE  FWGNGTPADR |  | >SCK05923.1 hypothetical protein [Streptomyces sp. AmelKG-E11A]  MSSGETALRPIDEELLLLTAYLLSSGRGLLEEPQQYGPFR  CIDAARRVLVLLRGRGVTNSELQELHGRLEDFMCGPMAP  RDLTAFLDEVCGKLTLLLRDSDLIRRGPASPATT |
| CDO-*Slau*31255 | *Streptomyces laurentii* strain ATCC 31255 |  | >BAU83480.1 nitroreductase [Streptomyces laurentii]  MDLSTSLASSSATLQVIRTRSVVRQFTGEPVDDDRLDALLDAMLAAPTGGNKQA  WAFVAVRDPRTLRVLRAFSPGLIEPSPLVVVACFDRSRAVKDDGEFWDEGLLCV  AMAVQNLLLAAHSMGLGGCPAASFRRTSVQALLRLPEHLEPLLLVPVGYPKRDL  VSPPRRDRSEVVGHDFWGNPSPTGR |  | >BAU83479.1 hypothetical protein [Streptomyces laurentii]  MISGETPPRPVDEELILLAAYLLSCGRGLLDEPQVYGTFRC  IDAARRVLVLLKDRGLDNSELLALHSQLEDFMCGPMIQR  DVTGFLDEVCLRLTLQLRDSDLIPQEPPSVVPV |

**Table S1.** Continued.

| **CDO name** | **Organism** |  | **CDOA and CDOB sequences (Fasta format)** | | |
| --- | --- | --- | --- | --- | --- |
|  |  |  | **CDOA** |  | **CDOB** |
| CDO-*Npot*45234 | *Nocardiopsis potens* DSM 45234 |  | >WP_051065445.1 nitroreductase family protein [Nocardiopsis potens]  MRVLLGRSAVRDFTPEAIDPEVLRRLLDAMIAAPSAGNVQAWAFVAVQDPRTL  KLLRAFAPGIIQPAPLIVAACFDRSRAVKDDGEFWDEGLLCVAMAVENLLLAA  HSEGLGGCPVASFRRDSVQEILELPKHLEPIILASIGHPARPLASPPRRDRSEVIRH  ETWGNRDAAHN |  | >WP_051065442.1 hypothetical protein [Nocardiopsis potens]  MKPGGTVTQPTTEEVTEELVLLATHLLNCARGLFNEPQA  YGPIRCLDAARRTFIIAEKAGLRDERLADIRARLDDFMCG  PMEYHDLSDFLDELCAALLTALKGSDVLSSAGGERG |
| CDO-*Sflo*2465 | *Streptomyces flocculus* strain NRRL B-2465 |  | >WP_051705784.1 MULTISPECIES: nitroreductase [Streptomyces]  MRTRSVVRDYAERPVAHESVEELLDAMLAAPTGGNLQAWAFVAIQNPRNLKL  LRAFAPGIIQPPPLIVAACFDRSRAVKNDGTFWDEGLLCVAMAVENLLLAAHS  MGLGGCPVASFRRESVQALLELPKHVEPLLLVPIGYPARPLASPPRRDRSEVISY  EFWGNHDATRG |  | >WP_055496574.1 hypothetical protein [Streptomyces flocculus]  MSSGETTTRPVAEELVLLAAYLLSCGRGLLEEPQAYGPLR  CLDAGRRVLTLVEQAGLHDERLSAIRARLDDCMCGPMEY  RDLPGFLDEVCGSLITALKDSELISASTGHQE |
| CDO-*Salb*13014 | *Streptomyces albus* strainNBRC 13014 |  | >WP_041968570.1 MULTISPECIES: nitroreductase family protein [Streptomyces]  MRTRSVVRDYAERPVARESVEELLDAMLAAPTGGNLQAWAFVAIQNPRNLKL  LRAFAPGIIQPPPLIVAACFDRSRAVKNDGTFWDEGLLCVAMAVENLLLAAHS  MGLGGCPVASFRRESVQALLELPKHVEPLLLVPIGYPARPLASPPRRDRSEVIS  YEFWGNHDATRG |  | >WP_016471397.1 MULTISPECIES: hypothetical protein [Streptomyces]  MSSGETTTRPVAEELVLLAAYLLSCGRGLLEEPQAYGPLR  CLDAGRRVLTLVEQAGLHDERLSAVRARLDDCMCGPME  YRDLPGFLDEVCGSLITALKDSELISASTGHQE |
| CDO-*Ssp*5123 | *Streptomyces* sp. NRRL F-5123 |  | >WP_052397370.1 nitroreductase [Streptomyces sp. NRRL F-5123]  MDAGPAGPAGPAGSADAVLRVLRSRSVVRQYTGRQVDDEVLEMLVSAMLA  APTASNKQAWAFVAVRERRTLRLLGAFAPGIIGTPPLVVAACFDRSRPVDERG  PGEGGWDMGLLCVAMAVENLLLAAHALGLGGCPVGGFREGPVRTVLRLPAH  LDPVLLVPVGHPAGPLRPTDRRDRNEVLRHDTWEQ |  | >WP_078862145.1 hypothetical protein [Streptomyces sp. NRRL F-5123]  MREELLLLAAFLLSSGRGLADEPAVYGQARCLDAARRTL  ALVEGLGGQDPAVTGLRTELEAFMTGPIGGCGDINTLLDS  ACDRLAEVLCDRGRDVDLLPRP |
| CDO-*Nalk*80379 | *Nocardiopsis alkaliphila* YIM 80379 |  | >WP_017602839.1 hypothetical protein [Nocardiopsis alkaliphila]  METPVEVLEPGLQEETSQAVRVLTTRRAVRSFSDRPVRDDLLAPLLDSMVAA  PSASNKQAWAFVIVREPRTLRLLHAFAPGIIERPPLIVVACFDRSRAVGGGAW  DEGMLCVAMAVQNLLLAAHALDLGGCPSASFRKTPVRRFLRLPRHLEPLLLV  SIGHPAQSPRTAPRRDRNEVIRHEQWG |  | >WP_083903352.1 hypothetical protein [Nocardiopsis alkaliphila]  MEEELLLLAAYLLSSGRGLLEEPRSYGPLRCLDAARRVLR  LRVEAGGGENPELTSLKERMDEVMCGAMVDRELDVLLD  ELCDRLAAVVEAPGVISA |
| CDO-*Nlis*13360 | *Nocardiopsis listeri* NBRC 13360 |  | >WP_084392381.1 nitroreductase family protein [Nocardiopsis listeri]  MEIPVEVPEPEPQDQVRRTLRVLNTRRAVRSFNDESVRDDLLDPLLDSMLAAP  SASNKQAWAFVVVRRPRTLRLIHAFAPGIIERPPLIVVACFDHGRSVGGGAWD  EGMLCVAMAVQNLLLAAHALELGGCPTASFRKSPVRRFLRLPQHLEPLLLVSI  GHPAPNPRTTAPRRDRNEVISHEHWG |  | >WP_084392383.1 hypothetical protein [Nocardiopsis listeri]  MDEELLLLAAYLISSGRGLLEEPRDYGPLRCLDAARRVLRL  RARAGGAQSPELTSLKERMDEVMCGAMVDRDLAALLDEL  CDRLAAVVEEPGAVTA |
| CDO-*Nha*l44410 | *Nocardiopsis halotolerans* DSM 44410 |  | >WP_017573922.1 nitroreductase family protein [Nocardiopsis halotolerans]  MRAFTAQRVEDSLLEPMLDAMLAAPSASNKQAWAFVAVRDPRTLRLLRAFS  PGIIELPPLVVAACFDRSRAVGGSGGSWWDEGMLCVAMAVENLLLAAHCLG  LGGCPSGSFRRGPVRRFLGLPDHLEPLLLVPIGHPARPLSSAPRRDRNEVVSHE  RWGS |  | >WP_017573921.1 hypothetical protein [Nocardiopsis halotolerans]  MSAGEADGRRVGEELLLITAYLLSSGRGLLEEPPQYGTFRC  LDAARRVLALAERTGPCHPELDALRAWMDDVMCGAMAD  HELDVLLDQLCDRLAAVLEDPDVISA |
| CDO-*Nsp*163 | *Nocardiopsis* sp. RV163 |  | >WP_082166760.1 nitroreductase family protein [Nocardiopsis sp. RV163]  MTTRRAVRAFTDRPVDDSLLAPMLDAMLAAPSASNKQAWAFVAVRERRRLR  LLRAFAPGIIELPPLVVAACFDRSRAVGGSGGSTDSRDSWDEGMLCVAMAVE  NLLLAAHCLGLGGCPSGSFRAGPVRMLLGLPEHLEPLLLVPIGHPARPLGPAPR  RDRNEVVSHERWGT |  | >WP_047869098.1 hypothetical protein [Nocardiopsis sp. RV163]  MSDGEPDVRRVGEELLLLAAYLLSSGRGLLDEPRQYGTFRC  LDAARRVLALAAGTGPHHPELDALRGRMDDVMCGPMGDH  ELDTLLDQLCDRLASVLEDPDVIPD |

**Table S1.** Continued.

| **CDO name** | **Organism** |  | **CDOA and CDOB sequences (FASTA format)** | | |
| --- | --- | --- | --- | --- | --- |
|  |  |  | **CDOA** |  | **CDOB** |
| CDO-*Nsp*639 | *Nocardiopsis* sp. CNS-639 |  | >WP_081643016.1 MULTISPECIES: nitroreductase family protein [Nocardiopsis]  MTTRRAVRAFADRPVDDSLLDPMLDAMLAAPSASNKQAWAFVAVRERRAL  RLLRAFSPGIIELPPLIVAACFDRSRAVGGSGNSTDSRGSRDSWDEGMLCVAM  AVENLLLAAHCLGLGGCPSGSFRRGPVRRLLGLPDHLEPLLLVPIGHPARPLA  PAPRRDRNEVVSHERWGT |  | >WP_019610347.1 MULTISPECIES: hypothetical protein [Nocardiopsis]  MSAGEPDVRQVGEELLLLAAYLLSSGRGLLDEPRQYGTFR  CLDAARRVLALAAGTGPHHPELEALRGRMDDVMCGPMG  DHELDTLLDQLCERLATVLEDPDVISD |
| Ndas_1146/1147 | *Nocardiopsis dassonvillei* DSM 43111 |  | >WP_071621715.1 nitroreductase family protein [Nocardiopsis dassonvillei]  MDTGSSEPDANRCPSQRSSHALQTLTTRRAVRAFADRPVDDSLLDPMLDAM  LAAPSASNKQAWAFVAVRERRALRLLRAFSPGIIELPPLVVAACFDRSRAVG  GSGNSTDSGDSWDEGMLCVAMAVENLLLAAHCLGLGGCPSGSFRRGPVRR  LLGLPDHLEPLLLVPIGHPARPLAPAPRRDRNEVVSHERWGT |  | >WP_013152195.1 MULTISPECIES: hypothetical protein [Nocardiopsis]  MSAGEPEVRQVGEELLLLAAYLLSSGRGLLDEPRQYGTFR  CLDAARRVLALAAGTGPHHPELDALRGRMDDVMCGPMG  DHELDTLLDQMCERLATVLEDPDVISD |
| CDO-*Nsyn*44143 | *Nocardiopsis synnemataformans* DSM 44143 |  | >WP_017566637.1 nitroreductase [Nocardiopsis synnemataformans]  MRAFADRPVDDSLLEPMLDAMLAAPSASNKQAWAFVAVREPRTLRLLRAF  SPGVIELPPLIVAACFDRSRAVGGSGASGASDASRDSWDEGMLCVAMAVEN  LLLAAHCLGLGGCPSGSFRPGPVRRFLGLPEHLEPLLLVPIGHPARPLAPAPR  RDRTEVVSHERWGD |  | >WP_017566636.1 hypothetical protein [Nocardiopsis synnemataformans]  MSAGETDVRRVGEELLLLAAYLLSSGRGLLDEPRQYGTFR  CLDAARRVLALAAGTGPHHPDLDALRGRMDDVMCGAMG  DHELDTLLDQLCERLAAVLEEPDVISD |
| CDO-*Ndas*13392 | *Nocardiopsis dassonvillei* NBRC 13392 |  | >WP_061081218.1 nitroreductase [Nocardiopsis dassonvillei]  MRAFTDRPVDDSLLEPMLDAMLAAPSASNKQAWAFVVVRERRTLRLLRAF  SPGIIELPPLVVAACFDRSRAVGGSGSSRESWDEGMLCVAMAVENLLLAAH  CLGLGGCPSGSFRAGPVRRLLGLPDHLEPLLLVPVGHPARPLAPAPRRDRTE  VVSHERWGD |  | >WP_061081217.1 hypothetical protein [Nocardiopsis dassonvillei]  MSAGETDARRVGEELLLLAAYLLSSGRGLLDEPRQYGTFR  CLDAARRVLALAAGTGPHHPDLDALRGRMDDVMCGAMD  DHELDTLLDQLCERLAAVLEDPDVISD |
| CDO-*Nxin*90004 | *Nocardiopsis xinjiangensis* YIM 90004 |  | >WP_017608305.1 nitroreductase [Nocardiopsis xinjiangensis]  MLSTDTGTRTEDRATPALRVLTTRRVVRSFDGRPVEDELLDPMLDAMLAA  PSASNKQAWAFVTIRRSRTLRLVRAFSPGIIEPPPLIVAACFDRSRAVGGGA  WDEGMLCVAMAVENLLLAAHALGLGGCPSASFRKSPVQRFLGLPDHLEPL  LLVPIGHPARPIESAPRRDRNEVISHDRWRG |  | >WP_017608304.1 hypothetical protein [Nocardiopsis xinjiangensis]  MTAGEADGPRTGEELLLLSAYLLSSGRGLLEEPREYGPLRC  LDAARRVLSLRADLGHGDHPGLPELRSRMEDVMCGAMAD  RELDVLLDDLCDRLASVLEGTDAVSA |
| CDO-*Nalb*43377 | *Nocardiopsis alba* DSM 43377 |  | >WP_042281961.1 nitroreductase [Nocardiopsis alba]  MLRVLTSRRVVRSFSDRPVDDETLEHLADAMVAAPSASNKQAWGFVMVR  RTRTLRMVHAFSPGIIERPPLVVAACFDRSRAVGGGGAWDEGMLCVAMAV  ENLLLAAHALGLGGCPSASFRKAPVRRFLGLPEHLEPLLLVSIGHPARPLEA  APRRDRNEVISHECWGN |  | >WP_017534687.1 hypothetical protein [Nocardiopsis alba]  MREELLLLAAYLLSSGRGLLQEPPSYGPLRCLDAARRVLAL  RDGLGGEESPALADLRASMDDVMCGAMTDRELDVLLDDL  CDRLAAVVEEPGAISA |
| CDO-*Nsp*L17 | *Nocardiopsis* sp. L17-MgMaSL7 |  | >WP_017544373.1 MULTISPECIES: nitroreductase [Nocardiopsis]  MAVAAEEPGSSAESRASQALHVLNTRTVVRSFSDRPVEDDLLTPLLDAMLA  APSASNKQAWAFVTVRRPRTLKLVHAFSPGIIERPPLVVAACFDRSRAVGG  GAWDEGMLCVAMAVQNLLLAAHALGLGACPSASFRKGPVSRFLGLPDHLL  PLLLVSIGHPDRTPEAAPRRGRNEVISHECWGE |  | >WP_026129216.1 hypothetical protein [Nocardiopsis sp. L17-MgMaSL7]  MSAGVNDDRVGEELVLLAAYLLSSGRGLLEEPAAYGPLRC  LDAARRVLSLRSRIGVPDSPELTDLRARMDDVMCGAMAD  RELDVLLDDLCDRLAAALEEPGAVSA |
| CDO-*Nsp*366 | *Nocardiopsis* sp. SBT366 |  | >WP_049572039.1 nitroreductase [Nocardiopsis sp. SBT366]  MAVAVEEPGSSAESRASQALHVLNTRTVVRSFSDRPVEDDLLNPLLDAML  AAPSASNKQAWAFVAVRRTRTLKLVHAFSPGIIERPPLVVAACFDRSRAV  GGGAWDEGMLCVAMAVQNLLLAAHALGLGACPSASFRKGPVSRFLGLP  DYLLPLLLVSIGHPDRTPETAPRRGRNEVISHECWGQ |  | >WP_049572043.1 hypothetical protein [Nocardiopsis sp. SBT366]  MSAGVNDDRVGEELVLLAAYLLSSGRGLLEEPAAYGPLRC  LDAARRVLSLRSRIGVADSPELTDLRARMDDVMCGAMAD  RELDVLLDELCDRLAAALEEPGAVSA |

**Table S2.** Characteristics of the CDPSs encoded in the pIJ196-CDPS plasmids used in this study.

| **Name** |  | **Alternate name in other studies** |  | **Organism** |  | **Major CDPs synthesized** |  | **Reference** |
| --- | --- | --- | --- | --- | --- | --- | --- | --- |
|  |  |  |  |  |  |  |  |  |
| **CDPSs used for expression with the associated CDO in the natural biosynthetic pathway** | | | | |  |  |  |  |
|  |  |  |  |  |  |  |  |  |
| CDPS2-*Aoli*43269 |  | no |  | *Actinomadura oligospora* ATCC 43269 |  | cWW |  | this study |
| CDPS1-*Aoli*43269 |  | CDPS66 |  | *Actinomadura oligospora* ATCC 43269 |  | cFY, cYY |  | [1] |
| CDPS-*Npot*45234 |  | CDPS62 |  | *Nocardiopsis potens* DSM 45234 |  | cFM, cFF, cFA, cFY, cFL |  | [1] |
| CDPS-*Said*5739 |  | DmtB3 |  | *Streptomyces aidingensis* strain CGMCC 4.5739 |  | cWL |  | [2] and this study |
| CDPS-*Scat*2342 |  | CDPS67 |  | *Streptomyces catenulae* strain NRRL B-2342 |  | cFY |  | [1] |
| CDPS-*Srim*3904 |  | CDPS69 |  | *Streptomyces rimosus* strain NRRL WC-3904 |  | cWY |  | [1] |
| CDPS-*S*sp5123 |  | CDPS75 |  | *Streptomyces* sp. NRRL F-5123 |  | cWP |  | [1] |
|  |  |  |  |  |  |  |  |  |
| **CDPSs used for expression in combinatorial engineering experiments with CDOs** | | | | |  |  |  |  |
|  |  |  |  |  |  |  |  |  |
| CDPS-*Blat* |  | CDPS37 |  | *Burkholderia lata* |  | cAE |  | [3] |
| CDPS-*Fdum* |  | CDPS29 |  | *Fluoribacter dumoffii* Tex-KL |  | cAA |  | [3] |
| CDPS-*Maer* |  | CDPS27 |  | *Micavibrio aeruginosavorus* EPB |  | cGN |  | [3] |
| CDPS-*Mmed*1 |  | CDPS13 |  | *Marinomonas mediterranea* MMB-1 |  | cLL |  | [3] |
| CDPS-*Mpro* |  | CDPS18 |  | *Moorea producens* |  | cAP |  | [3] |
| CDPS-*Othe* |  | CDPS8 |  | *Candidatus* Odyssella thessalonicensis L13 HMO |  | cGV |  | [3] |
| CDPS-*P*sp20_3 |  | CDPS17 |  | *Parabacteroides* sp. 20_3 |  | cHF |  | [3] |
| CDPS-*Pamo*25 |  | CDPS26 |  | *Candidatus* Protochlamydia amoebophila UWE25 |  | cPP |  | [3] |
| CDPS-*Ppro*5 |  | CDPS44 |  | *Pseudomonas protegens* Pf-5 |  | cLE |  | [3] |
| CDPS-*Rgry* |  | CDPS24 |  | *Rickettsiella grylli* |  | cFF, cFL |  | [3] |
| CDPS-*S*sp1868 |  | CDPS74 |  | *Streptomyces* sp. NRRL S-1868 |  | cWP |  | [1] |
| CDPS-*S*sp5053 |  | CDPS68 |  | *Streptomyces* sp. NRRL F-5053 |  | cWL |  | [1] |
| CDPS-*Scat*2342 |  | CDPS67 |  | Streptomyces catenulae strain NRRL B-2342 |  | cFY |  | [1] |
| CDPS-*Scat*8057 |  | CDPS14 |  | *Streptomyces cattleya* NRRL 8057 |  | cWW |  | [3] |
| CDPS-*Sesp*44229 |  | CDPS4 |  | *Saccharothrix espanaensis* DSM 44229 |  | cCC |  | [3] |
| CDPS-*Sjap* |  | CDPS10 |  | *Sphingobium japonicum* UT26S |  | cPM |  | [3] |
| CDPS-*Srim*3904 |  | CDPS69 |  | *Streptomyces rimosus* strain NRRL WC-3904 |  | cWY |  | [1] |
| CDPS-*Tvir* |  | CDPS101 |  | *Thalassomonas viridans* |  | cYY |  | [1] |
|  |  |  |  |  |  |  |  |  |

**Table S3**.NMR data for cYΔF, cΔYF, cWΔY, cWΔP, and cWΔL.

| **Compound** | **Produced by** | **Name and chemical structure** | **^1^H NMR (500.3 MHz, DMSO)** | **^13^C NMR (125.8 MHz, DMSO)** | **^15^N NMR (50.7 MHz, DMSO)** |
| --- | --- | --- | --- | --- | --- |
| **1** | CDPS1-*Aoli*43269 +  CDO1-*Aoli*43269 | cYΔF  ^^ | *δ* 9.17 (s, 1H, Hη Tyr), 8.44 (s, 1H, H^N^ ΔPhe), 8.38 (d, *J* = 2.7 Hz, 1H, H^N^ Tyr), 7.33 (m, 2H, Hε ΔPhe), 7.24 (m, 3H, Hδ + H ΔPhe), 6.94 (AA’XX’, 2H, Hδ Tyr), 6.60 (AA’XX’, 2H, Hε Tyr), 6.35 (s, 1H, Hβ ΔPhe), 4.26 (ddd, *J* = 5.1, 4.0, 2.7 Hz 1H, Hα Tyr), 3.03 (dd, *J* = 13.8, 4.0 Hz, 1H, Hβ Tyr), 2.83 (dd, *J* = 13.8, 5.1 Hz, 1H, Hβ' Tyr) | *δ* 166.2 (C' Tyr), 159.8 (C' ΔPhe), 156.3 (C Tyr), 133.3 (Cγ ΔPhe), 131.0 (Cδ Tyr), 128.9 (Cδ ΔPhe), 128.4 (Cε ΔPhe), 127.6 (C ΔPhe), 126.4 (Cα ΔPhe), 125.0 (Cγ Tyr), 114.9 (Cε Tyr), 113.2 (Cβ ΔPhe) 56.4 (Cα Tyr), 38.8 (Cβ Tyr); | * *133.2 (N ΔPhe), 114.1 (N Tyr) |
| **2** | CDPS1-*Aoli*43269 +  CDO1-*Aoli*43269 | cΔYF   | *δ* 9.53 (br s, 1H, Hη ΔTyr), 8.48 (s, 1H, H^N^ ΔTyr), 8.32 (d, *J* = 2.8 Hz, 1H, H^N^ Phe), 7.22 (m, 2H, Hε Phe), 7.17 (m, 2H, Hδ Phe), 7.13 (m, 1H, H Phe), 7.09 (AA’XX’, 2H, Hδ ΔTyr), 6.73 (AA’XX’, 2H, Hε ΔTyr), 6.27 (s, 1H, Hβ ΔTyr), 4.30 (td, *J* = 4.8, 2.8 Hz, 1H, Hα Phe), 3.11 (dd, *J* = 13.6, 4.4 Hz, 1H, Hβ Phe), 2.94 (dd, *J* = 13.6, 5.1 Hz, 1H, Hβ' Phe) | *δ* 165.9 (C' Phe), 160.4 (C' ΔTyr), 157.3 (C ΔTyr), 135.4 (Cγ Phe), 130.6 (Cδ ΔTyr), 130.0 (Cδ Phe), 128.1 (Cε Phe), 126.7 (C Phe), 123.9 (Cα ΔTyr), 123.9 (Cγ ΔTyr), 115.4 (Cε ΔTyr), 114.3 (Cβ ΔTyr) 56.2 (Cα Phe), 39.8 (Cβ Phe). |  |
| **3** | CDPS-*Srim*3904 +  CDO-*Srim*3904 | cW∆Y   | *δ* 10.83 (d, *J* = 2 Hz, 1H, Hε1 Trp), 9.56 (s, 1H, Hη ΔTyr), 9.20 (s,1H, H^N^ ΔTyr), 8.24 (d, *J* = 3.0 Hz, 1H, H^N^ Trp), 7.54 (m, 1H, Hε3 Trp), 7.17 (m, 1H, H2 Trp), 7.01 (m, 1H, Hη2 Trp), 7.00 (m, 2H, Hδ1 + 3 Trp), 6.54 (AA’BB’, 2H, Hε ΔTyr 6.50 (AA’BB’, 2H, Hδ ΔTyr, 6.15 (s, 1H, Hβ ΔTyr 4.22 (m, 1H, Hα Trp), 3.33 (m, 1H, Hβ Trp 3.01 (dd, *J* = 14.5, 4.7 Hz, 1H, Hβ’ Trp) | *δ* 166.6 (C’ Trp), 160.6 (C’ ΔTyr), 156.9 (C ΔTyr), 136.1 (Cε2 Trp), 130.2 (Cδ ΔTyr), 127.6 (Cδ2 Trp), 125.2 (Cδ1 Trp), 124.2 (Cα ΔTyr), 123.9 (Cγ ΔTyr), 120.8 (Cη2 Trp), 118.5 (Cε3 Trp), 118.3 (C3 Trp), 115.1 (Cε ΔTyr), 113.7 (Cβ yr 111.2 (C2 Trp), 107.4 (Cγ Trp), 56.1 (Cα Trp), 30.3 (Cβ Trp); | * *133.2 (Nε1 Trp + N ΔTyr), 114.1 (N Trp) |
| **4** | CDPS-*Ssp5*123 +  CDO-*Ssp5*123 | cW∆P   | *δ* 10.86 (s, 1H, Hε1 Trp), 8.29 (d, *J* = 2.4 Hz, 1H, H^N^ Trp), 7.46 (dd, *J* = 7.9, 0.8 Hz, 1H, Hε3 Trp), 7.28 (dt, *J* = 8.1, 0.9 Hz, 1H, H2 Trp), 7.03 (ddd, *J* = 8.1, 6.9, 1.2 Hz, 1H, Hη2 Trp), 6.94 (d, *J* = 3.5 Hz, 1H, Hδ1 Trp), 6.93 (ddd, *J* = 8.0, 6.9, 1.1 Hz, 1H, 3 Trp), 5.45 (t, *J* = 3.0 Hz, 1H, Hβ ΔPro 4.30 (dt, *J* = 4.4, 3.0 Hz, 1H, Hα Trp), 3.60 (td, *J* = 11.7, 7.7 Hz, 1H, Hδ ΔPro 3.39 (td, *J* = 11.8, 5.9 Hz, 1H, Hδ' ΔPro 3.31 (dd, *J* = 14.4, 3.3 Hz, 1H, Hβ Trp), 2.99 (dd, *J* = 14.4, 4.7 Hz, 1H, Hβ’ Trp), 2.33 (dddd, *J* = 18.1, 11.3, 6.0, 3.1 Hz, 1H, Hγ ΔPro 1.91 (dddd, *J* = 18.0, 11.4, 7.7, 2.9 Hz, 1H, Hγ' ΔPro | *δ* 162.8 (C’ Trp), 156.8 (C’ ΔPro), 136.0 (Cε2 Trp), 127.5 (Cδ2 Trp), 125.2 (Cδ1 Trp), 133.0 (Cα ΔPro), 120.8 (Cη2 Trp), 118.7 (Cε3 Trp), 118.1 (C3 Trp), 115.9 (Cβ ΔPro 111.1 (C2 Trp), 107.3 (Cγ Trp), 57.2 (Cα Trp), 44.9 (Cδ ΔPro 30.1 (Cβ Trp), 26.8 (Cγ ΔPro | *δ* 144.9 (N ΔPro), 131.9 (Nε1 Trp), 118.1 (N Trp) |
| **5** | CDPS-*Said*5739 +  CDO-*Said*5739 | cW∆L   | *δ* 10.85 (s, 1H, Hε1 Trp), 9.56 (s, 1H, H^N^ ΔLeu), 8.15 (d, *J* = 2.5 Hz, 1H, H^N^ Trp), 7.51 (d, *J* = 8 Hz, 1H, Hε3 Trp), 7.26 (dt, *J* = 8.0, 0.8 Hz, 1H, H2 Trp), 7.00 (ddd, *J* = 8.1, 7.0, 1.1 Hz, 1H, Hη2 Trp), 6.98 (d, *J* = 2.5 Hz, 1H, Hδ1 Trp), 6.92 (ddd, *J* = 7.9, 6.9, 1.1 Hz, 1H, 3 Trp), 5.18 (d, *J* = 10.3 Hz, 1H, Hβ ΔLeu 4.22 (td,  *J* = 4.3, 2.5 Hz, 1H, Hα Trp), 3.28 (dd, *J* = 14.5, 4.0 Hz, 1H, Hβ Trp), 3.00 (dd, *J* = 14.5, 4.7 Hz, 1H, Hβ’ Trp), 2.45 (m, 1H, Hγ ΔLeu 0.78 (d, *J* = 6.6 Hz, 3H, Hδ ΔLeu 0.52 (d, *J* = 6.6 Hz, 3H, Hδ' ΔLeu | *δ* 166.5 (C’ Trp), 159.5 (C’ ΔLeu), 135.9 (Cε2 Trp), 127.6 (Cδ2 Trp), 125.0 (Cα ΔLeu), 124.7 (Cδ1 Trp), 122.9 (Cβ ΔLeu 120.7 (Cη2 Trp), 118.6 (Cε3 Trp), 118.2 (C3 Trp),  111.0 (C2 Trp), 107.6 (Cγ Trp), 55.7 (Cα Trp), 29.7 (Cβ Trp), 23.3 (Cγ ΔLeu 22.3 (Cδ ΔLeu 21.9 (Cδ' ΔLeu | * *132.7 (Nε1 Trp + N ΔLeu), 114.6 (N Trp) |

**Table S4**.^3^J_Hβ-CO_ coupling constants and key ROEs observed for the dehydroaminoacids.

| Compound | Name | ^3^J_Hβ-CO_ (Hz) | ROE correlations |
| --- | --- | --- | --- |
| **1** | cY∆F | 5.3 | - |
| **2** | c∆YF | 5.3 | - |
| **3** | cW∆Y | 5.3 | ΔY HN / Hδ_1,2_ |
| **4** | cW∆P | 2.1 | - |
| **5** | cW∆L | 5.1 | ΔL HN / Hγ |

**Table S5**. Detection of CDPs and their derivatives in the supernatants of cultures of bacteria expressing CDPSs alone or in combination with diverse CDOs.

^a^CDPSs are named according to Table S2.

^b^The m/z values corresponding to CDPs are indicated; RT, retention time.

^c^Peak areas measured on extracted ionic current chromatograms are indicated in scientific notation (2.74E+09 means 2.74 × 10^9^); peak areas measured on UV chromatograms recorded at 214 nm are indicated in brackets; -, no detection; nm, not measurable because of coelution with another compound.

**Table S5**. Continued.

^a^CDPSs are named according to Table S2.

^b^The m/z values corresponding to CDPs are indicated; RT, retention time.

^c^Peak areas measured on extracted ionic current chromatograms are indicated in scientific notation (2.74E+09 means 2.74 × 10^9^); peak areas measured on UV chromatograms recorded at 214 nm are indicated in brackets; -, no detection; nm, not measurable because of coelution with another compound.

**Table S5**. Continued.

^a^CDPSs are named according to Table S2.

^b^The m/z values corresponding to CDPs are indicated; RT, retention time.

^c^Peak areas measured on extracted ionic current chromatograms are indicated in scientific notation (2.74E+09 means 2.74 × 10^9^); peak areas measured on UV chromatograms recorded at 214 nm are indicated in brackets; -, no detection; nm, not measurable because of coelution with another compound.

**Table S5**. Continued.

^a^CDPSs are named according to Table S2.

^b^The m/z values corresponding to CDPs are indicated; RT, retention time.

^c^Peak areas measured on extracted ionic current chromatograms are indicated in scientific notation (2.74E+09 means 2.74 × 10^9^); peak areas measured on UV chromatograms recorded at 214 nm are indicated in brackets; -, no detection; nm, not measurable because of coelution with another compound.

**Table S5**. Continued.

^a^CDPSs are named according to Table S2.

^b^The m/z values corresponding to CDPs are indicated; RT, retention time.

^c^Peak areas measured on extracted ionic current chromatograms are indicated in scientific notation (2.74E+09 means 2.74 × 10^9^); peak areas measured on UV chromatograms recorded at 214 nm are indicated in brackets; -, no detection; nm, not measurable because of coelution with another compound.

**Table S5**. Continued.

^a^CDPSs are named according to Table S2.

^b^The m/z values corresponding to CDPs are indicated; RT, retention time.

^c^Peak areas measured on extracted ionic current chromatograms are indicated in scientific notation (2.74E+09 means 2.74 × 10^9^); peak areas measured on UV chromatograms recorded at 214 nm are indicated in brackets; -, no detection; nm, not measurable because of coelution with another compound.

**Table S6**. Database information and sequence data relative to previously uncharacterized CDPSs.

| **CDPS name** |  | **Organism** |  | **NCBI entry and sequence (FASTA format)** |  | **Synthetic gene**^a,b^ |
| --- | --- | --- | --- | --- | --- | --- |
| CDPS2-*Aoli*43269 |  | *Actinomadura oligospora* ATCC 43269 |  | >WP_051468276.1 tRNA-dependent cyclodipeptide  synthase [Actinomadura oligospora]  MHEAAVERLTAPFTMTPYTENCARLSERAEHVL  VGVSPGNGYFNQERLTALLRWASATFGQVDAI  VPDASLVHTYQALGQSPETAWTNARHKVGKTY  RRVARAWAEIGVPPGEQRIHLLSDFVDHPVYTH  LRDETDRAVERDPALREAFLQTSRRVLSAFLKD  EEPTDEQVEEGKNYLTAEMPLCLDTPALLGVSS  SVAVYHHRLPMAEVMFSSPYLNVSPLQGHAVV  RPSEGGR |  | CCATGGCACATGAAGCCGCAGTTGAACGTTTAACCGCCCCTTTTACCATGACCCCGTATACCG  AGAATTGCGCACGTCTGAGCGAACGCGCAGAACATGTGCTGGTGGGTGTGAGCCCGGGTAAC  GGCTATTTTAATCAAGAACGTCTGACCGCTTTACTGCGTTGGGCAAGCGCCACCTTTGGTCAA  GTTGATGCAATTGTGCCGGACGCCAGTCTGGTGCATACCTATCAAGCTTTAGGCCAGAGTCCG  GAAACCGCATGGACCAATGCACGCCATAAGGTGGGCAAAACCTATCGCCGCGTTGCCCGTGC  ATGGGCCGAAATCGGTGTTCCGCCGGGCGAACAGCGCATTCATCTGCTGAGCGATTTCGTGGA  TCACCCGGTGTACACCCATCTGCGCGATGAAACCGATCGTGCCGTTGAACGCGATCCGGCACT  GCGCGAGGCCTTTCTGCAGACCAGCCGTCGTGTGCTGAGCGCCTTTCTGAAAGATGAGGAACC  GACCGATGAGCAAGTTGAAGAAGGTAAGAATTATTTAACCGCAGAAATGCCGCTGTGTCTGG  ATACCCCGGCACTGCTGGGCGTTAGCAGCAGCGTTGCCGTTTATCATCATCGTTTACCGATGG  CCGAGGTGATGTTTAGCAGCCCGTATCTGAACGTGAGCCCGCTGCAAGGTCATGCAGTTGTGC  GCCCTAGCGAGGGCGGTCGTAGATCT |
| CDPS-*Said*5739 |  | *Streptomyces aidingensis* strain CGMCC 4.5739 |  | >SFD40868.1 cyclo(L-tyrosyl-L-tyrosyl) synthase  [Streptomyces aidingensis]  MTAPAPRPATAFAVEPLTRNCRDVLVRGDHVLI  GVSPGNGYFTETRLTELLCWAAGAFRRIDVMIP  DCAEAETWIALGHTPEQARHKARAKARRVRNR  VTRAWAAAAVPAAGFGLHLLSEFTALPRYRAL  VRETERALTRDGGLHEGYRRAVHAALRAHLAG  ADPTPEQTRRAMRYLTAETPFLLDSPGLLDAAS  SVLVYHRRMEFLEPVFLGRTPLHPSRHQAFAVV  RPAGADDAGADAPGNGGDPA |  | CCATGGCAACCGCACCGGCCCCGCGTCCGGCAACAGCATTTGCAGTGGAACCGCTGACCCGC  AATTGCCGCGATGTTCTGGTGCGCGGTGATCATGTGCTGATTGGCGTTAGCCCGGGCAATGGC  TATTTTACCGAGACCCGTCTGACCGAACTGCTGTGTTGGGCCGCTGGTGCATTTCGCCGCATT  GACGTGATGATTCCGGATTGCGCCGAAGCAGAAACTTGGATCGCACTGGGCCATACACCGGA  ACAAGCTCGCCACAAAGCACGCGCCAAAGCCCGTCGCGTGCGTAATCGTGTGACCCGTGCTT  GGGCAGCAGCAGCAGTTCCCGCTGCTGGTTTTGGTTTACATCTGCTGAGCGAATTTACCGCTTT  ACCGCGTTATCGTGCTTTAGTTCGCGAAACCGAACGCGCATTAACCCGCGATGGCGGTTTACA  TGAAGGTTATCGTCGTGCCGTGCATGCCGCATTACGCGCCCATTTAGCCGGTGCAGATCCGAC  CCCGGAACAGACCCGTCGTGCCATGCGCTATCTGACCGCAGAAACCCCGTTTCTGCTGGATAG  TCCGGGTCTGCTGGACGCCGCCAGTAGTGTGCTGGTGTACCATCGCCGCATGGAGTTTCTGGA  ACCGGTTTTTCTGGGTCGTACCCCGCTGCATCCGAGCCGTCATCAAGCTTTTGCCGTTGTTCGT  CCCGCTGGTGCCGATGATGCTGGTGCAGATGCACCCGGTAACGGCGGTGATCCGGCAAGATCT |

^a^The 5’ and 3’ cloning sites *Nco*I and *Bgl*II are shown in red.

^b^A GCA codon (underlined) encoding an alanine was added after the start codon for cloning.

**Table S7**. Sequences of the genes encoding CDOs.

| **CDO name** |  | **Organism** |  | **Sequence of the CDO gene**^a,b^ |
| --- | --- | --- | --- | --- |
| CDO2-*Aoli*43269 |  | *Actinomadura oligospora* ATCC 43269 |  | ccatggacacgaccgacaccgctcctccggcgccggccgcgccacaggcgacgtccgcgccaccggcgccgccgcccgccgaacacgccgtcgaccccgccggggtcctccaggtgatccggacgcgcgccgtcgtccgcgagttcaccggcgagcccgtctcggacgacgacgccaaggccctcgccgccgccttggtggctgcgcccaacggcgcgaacctgcaggcgtgggcgttcgtcatcgtccggaggcctcagctcctgctctcggtccgcgccttcgcgcccggcgtgttcgcgatcccggcgctcatcctggtcgcctgtctcgaccactcccgcgccgccgacgagagcgacacccggacccacaaagagggcaggttgtgcgtggcgatggccgtggagaacttcctgctggcggcgcacgcgctcggcctgggcgcctgcccgtcctccagcttccttcccggcccgatccgggtgctgctggatctgcccgaccatctcgaacccgtcatggtggtctccgccggtcacccggcccacccgccgaaacccgctccgcgccgcgacatcgacgaggtcgtccactATGACAACTACGCCTGAGTCCACCCGCCGGGAGGCCGGTCTCAGCCGCCGGGAATCCGAACTCGCCCAGCTGGCCGCCTATCTCCTGGCCAGCAGTCGTTCGCTCCTGGACGACCCCGCGGTCTACGGCTCCTTCCGCCTCATCGACGCGGCCCGCCGGACGCTGCTCATCCTGGAGTCCGAGGGCGTCGCCAACGCCGACTTCACCGCCGTCCGAACCCAGATCGAAGAGGTCGTCCGCGCCAAGACCGAGGTCGACGTCCAGGCCTTCCTCGACACCCTGTGCCTTCAGATGGCCCACGCCCTCAAGGCCGCCGATCCCGACACCCTCGACACCAACCTCTGActcgag |
| CDO1-*Aoli*43269 |  | *Actinomadura oligospora* ATCC 43269 |  | ccatggccgggcgatcaacggacgagccgcagcgcgagcagcccgaggcggtacgggtactgctctcgcgcaccgccgtccgccagttcacggaccagcccgtcggcgacgacctcgtggggccgatgagcgaggcgttggtggccgcgccctcggcctcgaaccggcaggcatgggccttcgtcctcgttcgcgaccgacgcgtggtccgcctagtccaggcgttctcgccgggtgtcctcgcgacgcccccgctgatcgtcgtggcctgcttcgaccgttcccgcggcgtcggcgacaccggtgagcgctacgacgaatgtctgctgtgcgtggcgatggccgtacagaacctcctgctcgcggcccacgccctcggcctgggcggctgccccgtcgccagcttccgcgagaggccgctgcggcgggtgctcggcctgcccgcgcacatcgacccgatcctgctcgtctccgtcggacatcccgtccggctgaacccgcatcccgtccgccgcgatccgagtgaggtgatccaccATGACGTCTGGAGCGGCGGCGACGCCCCGCGAGCTCGGTGAGGAGCTCCTCCTGCTGGCCGCCTACCTGCTCAGCAGCGGCCGGGGCCTGTTCGACGAACCGCCCGCCTACGGTCCGCTCCGGTGCGCCGACGCGGCACGCCGCGTCCTCGGGCTCGTGGAACGGGCCGGAATCGAGCACCCGGAGATCCACGCCCTGCGCGCCGAGCTGGACGAGCTGTTCTTCGGTCCTATGGGCGACGGGAACATCCGCGAACTGCTCGACCTTCTGTGTGAGAGAACGGGAGCGCTGCTCCATGAGTCCGATGTCATCCAGACCTCCGGCGAATGActcgag |
| Ndas_1146/1147 |  | *Nocardiopsis dassonvillei* DSM 43111 |  | ccatggacacaggttcgagcgagccggatgcgaaccggtgcccctctcagcggtcatcacacgccctacagaccctgaccacccgccgtgccgtacgcgccttcgccgaccggccggtggacgactccctcctcgaccccatgctggacgccatgctcgccgccccctcggcgtccaacaagcaggcgtgggccttcgtcgccgtccgcgagcggcgggcgctgaggctgctgcgcgccttctcccccggaatcatcgaactcccgcccctggtcgtggcggcctgcttcgaccgctcccgtgccgtggggggctcaggcaactccacggactccggggactcctgggacgagggcatgctctgcgtcgcgatggcggtggagaacctcctcctggcggcccactgcctggggctgggcggatgcccgtccgggagctttcggaggggccccgtccgcaggctcctgggcctgcccgaccacctggaacccctgctcctggttccgatcgggcaccccgcccggccactcgcacccgcaccccgacgagaccggaacgaggtggtcagccATGAGCGCTGGGGAACCTGAGGTCCGACAGGTCGGCGAGGAACTCCTCCTGCTCGCCGCCTACCTGCTCAGCAGCGGCCGCGGCCTGCTGGACGAGCCACGGCAGTACGGCACGTTCCGCTGCCTGGACGCCGCCCGGCGCGTCCTCGCCCTCGCGGCCGGAACCGGCCCGCACCACCCCGAACTCGACGCCCTGCGCGGTCGGATGGACGACGTCATGTGCGGGCCGATGGGCGACCACGAACTGGACACCCTGCTCGACCAGATGTGCGAGCGGCTGGCAACCGTCCTGGAGGATCCCGATGTCATCTCCGACTGActcgag |
| CDO-*Npot*45234 |  | *Nocardiopsis potens* DSM 45234 |  | ccatggcaacgctccccgaccagccatccgacttcatgcgcgtcctgctcggcagaagcgccgtcagggacttcaccccggaggcgatcgatccggaggtgctgcggcgcctgctggacgccatgatcgccgccccgtccgccggcaacgtccaggcctgggccttcgtggccgtccaggaccctcgcacgctgaagctcctgcgggccttcgcgcccggcatcatccagccggcgccgctgatcgtcgccgcctgcttcgaccggtcccgtgccgtgaaggacgacggcgaattctgggacgaagggctgctgtgcgtggcgatggcggtggagaaccttctcctcgccgcgcattccgaggggctcggcggctgcccggtcgccagtttccgccgcgattccgtgcaggaaatccttgagctgccgaaacacctggagcccatcatcctggcgtccatcgggcatccggcgcgccccctggcgtcgccgccccgccgcgaccgcagcgaggtgatcagacATGAAACCTGGGGGAACCGTGACGCAGCCCACAACTGAAGAAGTCACCGAGGAATTGGTCCTGCTCGCGACGCATCTGCTCAATTGTGCACGCGGCCTTTTCAATGAACCGCAGGCCTATGGGCCCATACGCTGCCTGGACGCGGCGCGCAGGACTTTCATCATCGCCGAGAAGGCCGGCCTCCGCGACGAGCGGCTCGCCGATATCCGAGCACGATTGGACGACTTCATGTGCGGGCCGATGGAATACCACGACCTCTCCGACTTCCTGGACGAGCTCTGCGCGGCCCTGCTCACCGCGCTGAAGGGGTCGGACGTCCTCTCCTCGGCCGGCGGGGAGCGCGGATGActcgag |
| CDO-*Said*5739 |  | *Streptomyces aidingensis* strain CGMCC 4.5739 |  | ccatggagccgaccggtgagggcgaggaggtgctgcgggtgctgcgcggccgggcggtggtccgccgctacaccgccgagccggtggacgacgcactgctggaccggctgctggaggtcacggtcaccgcccccacggcggccaacaagcaggcgtggggctttctcgcggtgcgcgctccgcgcatggtgcggtgcctgcgcgccttcgcacccggcatgatcggcctgccgccgctggtggtggtggcgtgcttcgaccgcgaccgggcggtcggcgagccggccgccccctgggacgcgggcatgctgtgcgtcgctatggcggtggagaatctgctgctggccgcgcatgccgtgggcctgggcggctgtccggtgagcagcttcgaccgcgcggcggtgcggcgcctggtgcggctgccgccggccctggaaccgctgctgctggtgccgctcggacggcccgcccgcatcccggagccggccgcccgccgggagcgcagcgaggtgatcagatATGAGTCATGGGACTGAGCCTCCGGTGCCTCCGGTGCCGCCGGGGCCGCCGGCGTCCGCGGCGGCCGAGGGGCTGACCCTGCTGGCCGCCTTTCTGCTGAGCAGCGCCCGGGGCCTGCTCAACGAGCCCCCCGGCTACGGGGTGGCACGCTGCGCCGACGGGGCCCGCCGCACCCTGGAACTGCTGGACCTCTGCGGCGGCGGACCCGACCCGCGCCTGGTCCGCGTCCGGGAGCGGCTGGAGGAGACCATGTGCGGCCCGATGAGCGCGGCGGACTTCCCCGCCCTGCTGGACCGGGCGCTGGACGAGGTGGTGTCCGTGATCGAGGAGGGCGGGGACCGGGAGCCCGGCGGCCTCACCGGAGCGGGCTGActcgag |

^a^The *Nco*I and *XhoI* cloning sites are shown in red.

^b^The sequence of the CDOB-encoding gene is shown in upper case.

**Table S7**. Continued.

| **CDO name** |  | **Organism** |  | **Sequence of the CDO gene**^a,b^ |
| --- | --- | --- | --- | --- |
| CDO-*Scat*2342 |  | *Streptomyces catenulae* strain NRRL B-2342 |  | ccatgggaacggccgggccggggccgggcgtacggatcgccgcggcccgtggcgcggagacgctggacgtcatccgcacccgcggggtcgtgcggtcctacacggacgagccggtcggtgacgaactgctcgactcgttgctggaggcgatgctggcggccccctccgcctccaacaagcaggcgtgggcgtttctggcggtccgggcgccggaacgggtgcggcggctgcgggcgttcgcgccggggatcatcggggtgccgcccctcgtactggtcgcctgcgtcgaccacgcgcggatgacgggcgatccccacctacgggacgtcggctcgctctgtgtggcgatggcggtggagaacttcctgctcgccgcgcatgcgcaggggctgggcggctgcccggtgagcagcttcctggccgagcccgtacagctgctcctggggctgccgggccacctcgaacctcttctgctggtcccggtcggccgacccgaccagccccttcagccctcgccccgccgcgccccacaggaggtggtccgccATGAGTACTGGCACCCCACGCACCCCGGATGACCCGGGCGGGGAGGAGTTGGCGTTGCTCGCCGCCTACCTCCTCAGCAGCGCCCGGCGGCTGCTCCAGGAACCGCCGTCGTACGCGCTCTACCGGCTGATGGACGGCGCCCGCCGCGTCCTCGCGCTGTACGCGGACGGCGGTGGCGACCGTCCCGAACTGGTCGCGGTCCACGCCGGGCTGGACGACCTGCTGCACCAGGCGCCCAACGAGAACCGCGACTACGCGGCCCTGCTCGACGGGATGTGCCGGCAGATGGTCGCCGGTCTCCAACTGCCCGCGCCGGAGCCCGCCGCGTCCGCGCCGCCGTCCTGActcgag |
| AlbA/B |  | *Streptomyces noursei* ATCC 11455 |  | ccatggcattagctcacagttcatctgaatcgccgccggaatccttgccggacgcgtggacggtcctcaaaacccgtaccgccgtccgcaattacgcgaaagagccggtcgacgacgcgctgatcgagcagctgttggaggccatgctcgccgcgccgaccgcctccaaccggcaggcgtggtcgttcatggtggtgcgcaggcccgccgcggtccgccggctgcgcgcgttctcgcccggggtgctgggaacccccgccttcttcgtcgtggcctgcgtcgaccgcagtctgaccgacaacctctccccgaagctctcgcagaagatctacgacaccagcaagctctgtgtcgcgatggcggtggagaacctgctgctcgcggcgcacgcggccggcctgggcggatgcccggtgggcagcttcaggtccgacatcgtcaccagcatgctcggtatcccggaacacatcgagccgatgctcgtggtcccgatcggccgtcccgcgacagccctcgtcccctcccagcgccgcgccaagaatgaggtcgtcaactatgaatcctggggaaaccGTGCTGCCGCCCCAACTGCGTGAGGAGATCGCGCTCCTCGCCGTCTATCTGCTCAGCAGCGGCCGCGGACTCCTGGAGGAGCCGGCCGACTACGGAATTTACCGCTGTACCGACGGGGCCCGTCGGGCGCTCCAACTCCTCGACGAACACGGCGGGAGCACGGCACGGCTGACCGCCGTCCGCGAGCGTCTCGACGAGGTCATGTTCGCGCCGATGGGCGAGGACCGGGACATGGGCGCGATTCTGGACGACCTGTGTCGCCAAATGGCAGACGCTCTTCCGGAAATTGAAACCCCCTGActcgag |
| CDO-*Srim*3904 |  | *Streptomyces rimosus* strain NRRL WC-3904 |  | ccatggcactcagccaggagaccgccccggaggcgaccggttccgcgacgacggcagcggcgttcttgcgcctgttgcgctcccgggcggtggtgcgccgctacaccgacgagccggtggccgacgcgctgctggcagagctgctggacgtgatgccgacggcgcccaccgccgccaacaagcaggcgtgggcgttcgtggccgtccgggacccgtacgccgtgcgctgtctgcgggccttcgcaccgggcatgatcggcctgccgccgctggtggtggcggcctgtttcgaccgcgaccgcgccgtacgcgaggacagcggctcccaggacaccgggctgctgtgcgtggcgatggccgtgcagaacctgctcctggccgcgcacgcgtcgggcctgggcggttgccccgtcagcagcttctcgcgcaccgccgtgcaccgcctgctcgccctgccgcaccacctcgaacccgtcctgctcgtaccggtcggccacccggccgccccggtacgcccctccgcccgacgcgaccgcgacgaggtgatccaccATGACGTCTGGACCTCCCCCACCGCCGCGTCAGCCGGACCTTGACGATGACATCGTCCTGCTGGCCGCCTTCCTCCTGAGCAGCGGCCACGGCCTGCTGGACGAGCCCCCGGCCTACGGCCCGGCCCGCTGCGCCGACGGCGCCCGCCGCGCCCTGGAACTGCTCGACACGCACGGCACCCCGGACCCGGCCCTGACGCGGGTCCGCGAGCAGTTGGAGAACGCGATGTGCGGCTGCATGGCCGACGTCGACCTCCCCTCCCTGCTCCGGACCACCTGCGATCAGGTCCTGGACGTGGTCATGGCCCGCCGGGCGGGCGCTTCCCGCCTCCTGTGActcgag |
| CDO-*Ssp*5123 |  | *Streptomyces* sp. NRRL F-5123 |  | ccatggacgcggtggacgcgggtccggcaggtccggcgggtccggcgggctcggcggacgcggtgctgcgcgtactgcggagccgaagtgtcgtcaggcagtacaccggccggcaggtcgacgacgaggtcctggagatgctcgtgagcgcgatgctcgcggcgcccacggcctccaacaagcaggcgtgggccttcgtcgccgtgcgcgagcggcggacgctgcgcctgctcggcgcgttcgcccccgggatcatcggcacgccgccgctcgtcgtcgccgcctgtttcgaccggtcccggccggtcgacgaacgcgggcccggggaaggcggctgggacatggggctgctgtgcgtggcgatggccgtggagaacctgctgctggcagcccacgccctcgggctcggcggctgccccgtcggcggcttccgcgaggggccggtgcggacggtcctccggctccccgcacacctcgaccccgtgctgctggtgccggtcggccaccccgccgggccgctgcgcccgaccgaccgacgtgaccggaacgaggtgctcagacATGACACATGGGAGCAGTGACAGCGGCCGTATGCGGGAGGAACTGCTGCTGCTGGCGGCCTTCCTGCTCAGCAGCGGGCGCGGACTGGCGGACGAGCCGGCCGTGTACGGCCAGGCGCGGTGCCTGGACGCCGCCCGGCGCACGCTGGCCCTTGTCGAGGGGCTGGGCGGGCAGGACCCGGCCGTCACCGGGCTGCGCACCGAGCTGGAGGCGTTCATGACCGGGCCGATAGGCGGCTGCGGGGACATCAACACGCTGCTGGACTCGGCGTGCGACCGCCTCGCGGAGGTGCTGTGCGACCGGGGACGGGACGTGGACCTGCTCCCGCGCCCGTGActcgag |

^a^The *Nco*I and *XhoI* cloning sites are shown in red.

^b^The sequence of the CDOB-encoding gene is shown in upper case.

**Table S8.** Oligonucleotides and PCR conditions for the construction of pIJ194-CDO-*Snou*11455.

| **PCR** | **Oligonucleotides (5’ to 3’)^a,b^** | **PCR conditions** |
| --- | --- | --- |
| PCR1 | O1: AATGCTTACTGTTGCCCATGGCATTAGCTCACAGTTCATCTG  O2: GTTCTCCACCGCCAT**C**GCGACACAGAGCTTG | 98°C 2 min; 30 cycles of 98°C 10 sec, 61°C 15 sec, 72°C 30 sec ; 72°C 10 min. |
| PCR2 | O3: CAAGCTCTGTGTCGC**G**ATGGCGGTGGAGAAC  O4: GCACGTATCCTTTTGGCTCGAGTCAGGGGGTTTCAATTTC | 98°C 2 min; 30 cycles of 98°C 10 sec, 61°C 15 sec, 72°C 30 sec ; 72°C 10 min. |
| PCR3 | O1: AATGCTTACTGTTGCCCATGGCATTAGCTCACAGTTCATCTG  O4: GCACGTATCCTTTTGGCTCGAGTCAGGGGGTTTCAATTTC | 98°C 2 min; 30 cycles of 98°C 10 sec, 61°C 30 sec, 72°C 30 sec ; 72°C 10 min. |

^a^The restriction sites *Nco*I and *Xho*I are underlined

^b^The mutation introduced to eliminate the internal *Nco*I site in CDOA-*Snou*11455 is shown in bold.

**Table S9.** HPLC conditions for the purification of 2,5-DKPs.

| **Expressed recombinant proteins** |  | **HPLC gradient** |  | **Retention time** |  | **Purified compound** |
| --- | --- | --- | --- | --- | --- | --- |
| CDPS1-*Aoli*43269  CDO1-*Aoli*43269 |  | 0 min: 20% solvent B in A  5 min: 20% solvent B in A  20 min: 35% solvent B in A |  | 13.3 min (major product) |  | cY∆F |
|  |  |  |  | 14.9 min (minor product) |  | cF∆Y |
| CDPS-*Said*5739 |  | 0 min: 30% solvent B in A  5 min: 30% solvent B in A  14 min: 39% solvent B in A |  | 8.3 min |  | cWL |
| CDPS-*Said*5739  CDO-*Said*5739 |  | 0 min: 29% solvent B in A  5 min: 29% solvent B in A  19 min: 43% solvent B in A |  | 10.9 min |  | cW∆L |
| CDPS-*Srim*3904  CDO-*Srim*3904 |  | 0 min: 23% solvent B in A  5 min: 23% solvent B in A  13 min: 31% solvent B in A |  | 7.8 min |  | cWY |
|  |  |  |  | 9.4 min |  | cW∆Y |
| CDPS-*S*sp5123 |  | 0 min: 29% solvent B in A  5 min: 29% solvent B in A  19 min: 43% solvent B in A |  | 6.4 min |  | cWP |
| CDPS-*S*sp5123  CDO-*S*sp5123 |  | 0 min: 23% solvent B in A  5 min: 23% solvent B in A  13 min: 31% solvent B in A |  | 8.4 min |  | cW∆P |

**References for Additional file 1**

1. Gondry M, Jacques IB, Thai R, Babin M, Canu N, Seguin J, et al. A comprehensive overview of the cyclodipeptide synthase family enriched with the characterization of 32 new enzymes. Front Microbiol. 2018;9:46.

2. Yao T, Liu J, Liu Z, Li T, Li H, Che Q, et al. Genome mining of cyclodipeptide synthases unravels unusual tRNA-dependent diketopiperazine-terpene biosynthetic machinery. Nat Commun. 2018;9:4091.

3. Jacques IB, Moutiez M, Witwinowski J, Darbon E, Martel C, Seguin J, et al. Analysis of 51 cyclodipeptide synthases reveals the basis for substrate specificity. Nat Chem Biol. 2015;11:721–7.
