# Supplementary material for: In vivo characterization of the activities of novel cyclodipeptide oxidases: new tools for increasing chemical diversity of bioproduced 2,5-diketopiperazines in Escherichia coli
Source: Microb Cell Fact. 2020 Sep 7;19:178. doi: 10.1186/s12934-020-01432-y (PMC7487605; doi:10.1186/s12934-020-01432-y)
Supplement: Supplementary file 2 — Additional file 2: Fig. S1. Mass spectra of predicted ΔcFY (m/z 309). Fig. S2. 1D 1H NMR spectrum (with presaturation of residual water) and 1D 13C NMR DEPTQ spectrum of cYΔF in DMSO-d6. Fig. S3. 2D 13C-1H HSQC spectrum and 2D 13C-1H HMBC spectrum of cYΔF in DMSO-d6. Fig. S4. 1D 1H NMR spectrum (with presaturation of residual water) and 1D 13C NMR DEPTQ spectrum of cΔYF in DMSO-d6. Fig. S5. 2D 13C-1H HSQC spectrum and 2D 13C-1H HMBC spectrum of cΔYF in DMSO-d6. Fig. S6. 1D 1H NMR spectrum and 1D 13C NMR DEPTQ spectrum of cWΔY in DMSO-d6. Fig. S7. 2D 13C-1H HSQC spectrum and 2D 13C-1H HMBC spectrum of cWΔY in DMSO-d6. Fig. S8. 1D 1H NMR spectrum and 1D 13C NMR DEPTQ spectrum of cWΔP in DMSO-d6. Fig. S9. 2D 13C-1H HSQC spectrum and 2D 13C-1H HMBC spectrum of cWΔP in DMSO-d6. Fig. S10. 1D 1H NMR spectrum and 1D 13C NMR DEPTQ spectrum of cWΔL in DMSO-d6. Fig. S11. 2D 13C-1H HSQC spectrum and 2D 13C-1H HMBC spectrum of cWΔL in DMSO-d6. Fig. S12. Genomic environment of the CDO genes and predicted biosynthetic pathways. [file 12934_2020_1432_MOESM2_ESM.pdf]

Additional File 2

In vivo characterization of the activities of novel cyclodipeptide oxidases: new tools for increasing chemical diversity of bioproduced 2,5-diketopiperazines in *Escherichia coli*

Fabien Le Chevalier, Isabelle Correia, Lucrèce Matheron, Morgan Babin, Mireille Moutiez, Nicolas Canu, Muriel Gondry, Olivier Lequin, Pascal Belin

Fig. S1. Mass spectra of predicted ΔcFY (*m*/*z* 309).
Fig. S2. 1D ^{1}H NMR spectrum (with presaturation of residual water) and 1D ^{13}C NMR DEPTQ spectrum of cYΔF in DMSO-d_6.
Fig. S3. 2D ^{13}C-^{1}H HSQC spectrum and 2D ^{13}C-^{1}H HMBC spectrum of cYΔF in DMSO-d_6.
Fig. S4. 1D ^{1}H NMR spectrum (with presaturation of residual water) and 1D ^{13}C NMR DEPTQ spectrum of cΔYF in DMSO-d_6.
Fig. S5. 2D ^{13}C-^{1}H HSQC spectrum and 2D ^{13}C-^{1}H HMBC spectrum of cΔYF in DMSO-d_6.
Fig. S6. 1D ^{1}H NMR spectrum and 1D ^{13}C NMR DEPTQ spectrum of cWΔY in DMSO-d_6.
Fig. S7. 2D ^{13}C-^{1}H HSQC spectrum and 2D ^{13}C-^{1}H HMBC spectrum of cWΔY in DMSO-d_6.
Fig. S8. 1D ^{1}H NMR spectrum and 1D ^{13}C NMR DEPTQ spectrum of cWΔP in DMSO-d_6.
Fig. S9. 2D ^{13}C-^{1}H HSQC spectrum and 2D ^{13}C-^{1}H HMBC spectrum of cWΔP in DMSO-d_6.
Fig. S10. 1D ^{1}H NMR spectrum and 1D ^{13}C NMR DEPTQ spectrum of cWΔL in DMSO-d_6.
Fig. S11. 2D ^{13}C-^{1}H HSQC spectrum and 2D ^{13}C-^{1}H HMBC spectrum of cWΔL in DMSO-d_6.
Fig. S12. Genomic environment of the CDO genes and predicted biosynthetic pathways.

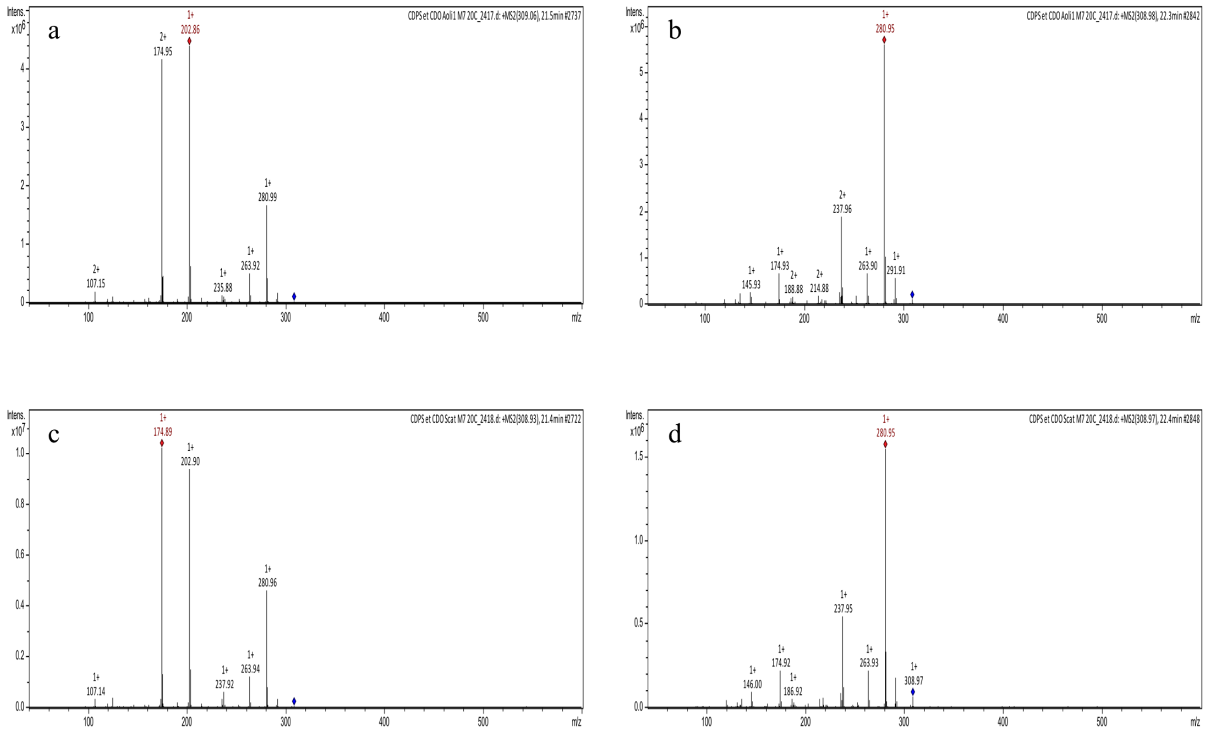

Fig. S1. Mass spectra of predicted ΔcFY (m/z 309). MS2 spectra were obtained from LC-MSMS analysis of SPE-treated culture supernatants of bacteria co-expressing either CDPS1 and CDO1 from *A. oligospora* (a and b) or CDPS and CDO from *S. cattleya* (c and d). Panels a and c correspond to compounds with a RT of 21.4 min, and panels b and d correspond to compounds with a RT of 22.3 min.

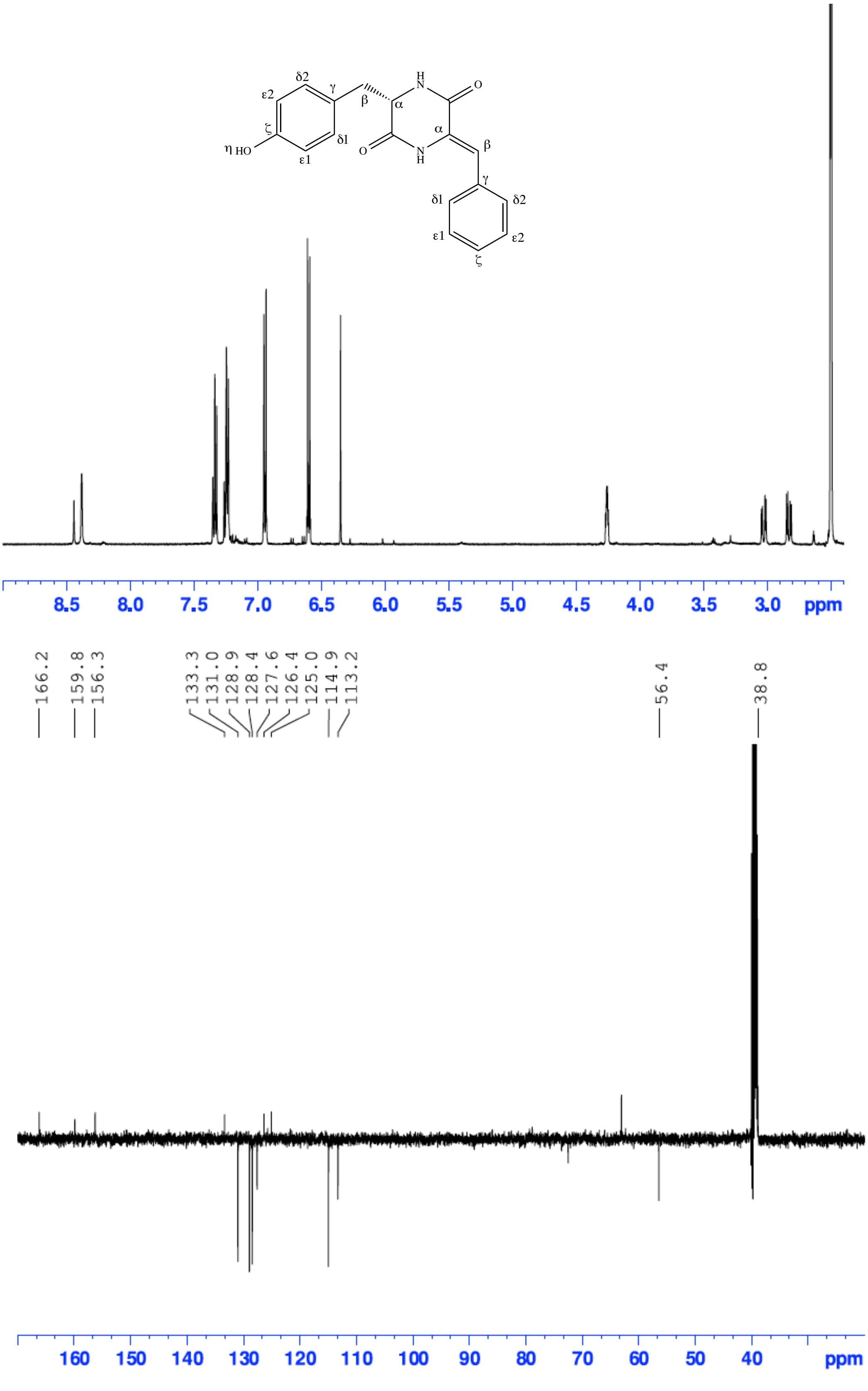

Fig. S2. 1D ^{1}H NMR spectrum with presaturation of residual water (top) and 1D ^{13}C NMR DEPTQ spectrum (bottom) of cYΔF in DMSO-d_6.

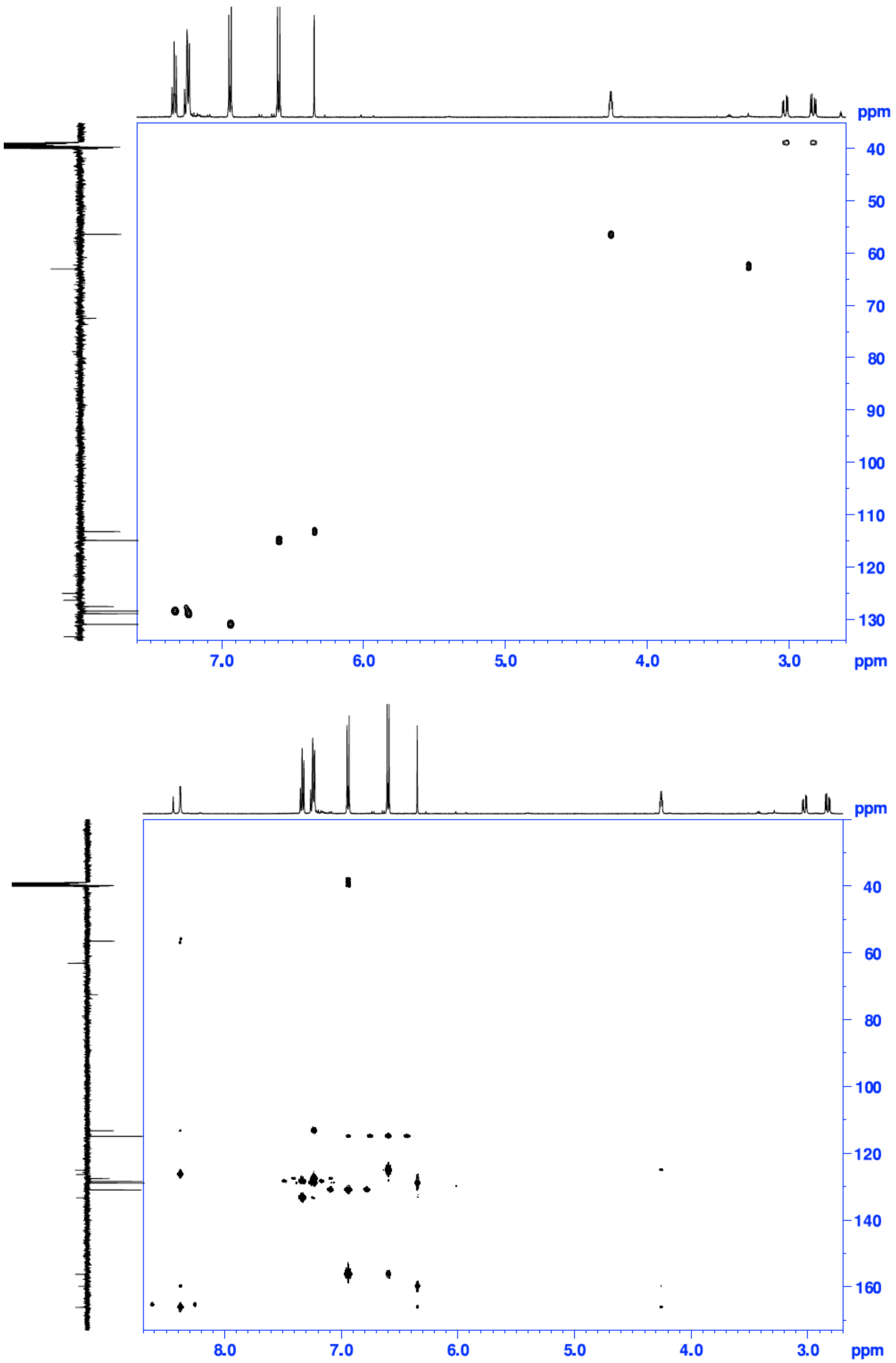

Fig. S3. 2D ^{13}C-^{1}H HSQC spectrum (top) and 2D ^{13}C-^{1}H HMBC spectrum (bottom) of cYΔF in DMSO-d_6.

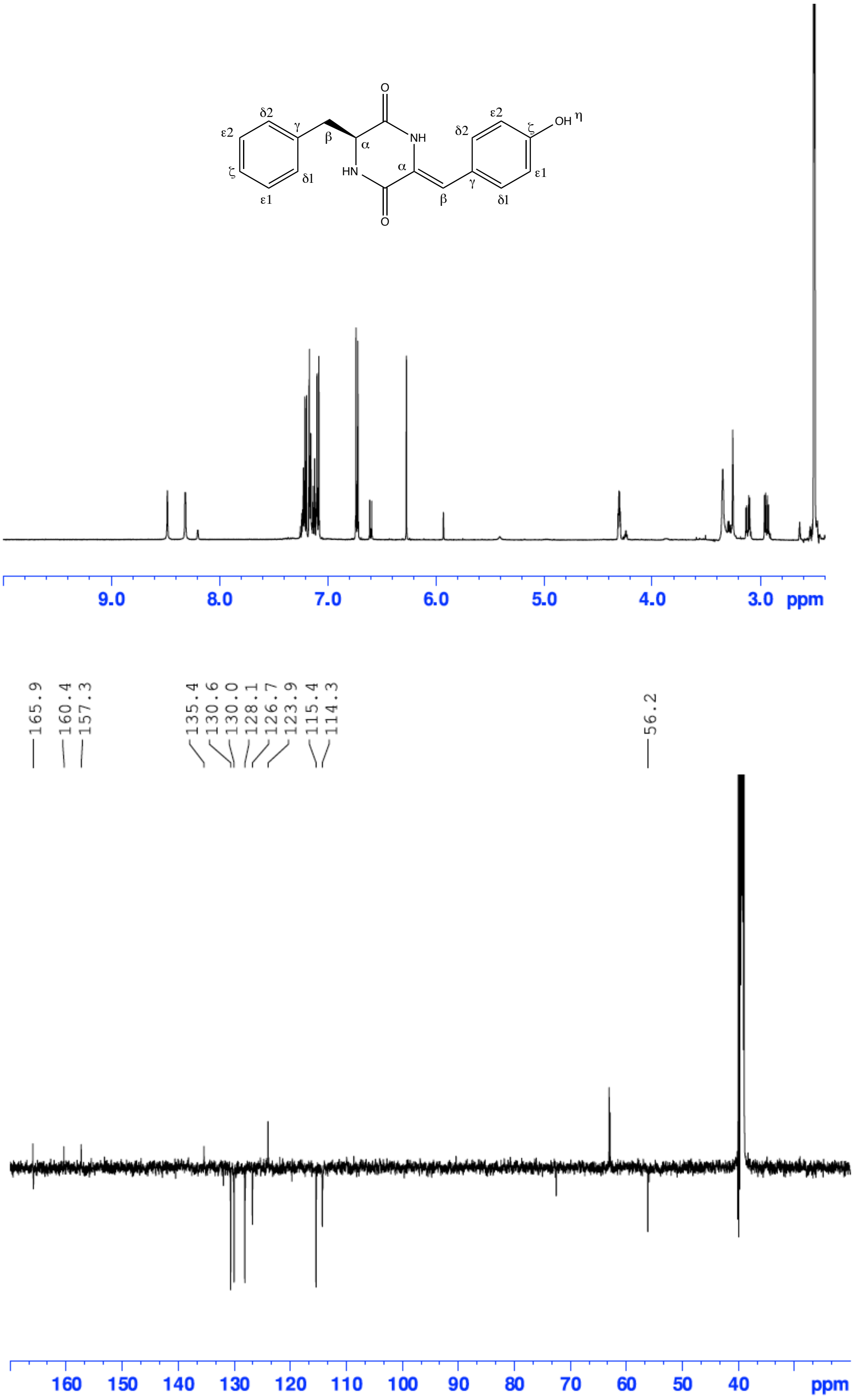

Fig. S4. 1D ^{1}H NMR spectrum with presaturation of residual water (top) and 1D ^{13}C NMR DEPTQ spectrum (bottom) of cΔYF in DMSO-d_6.

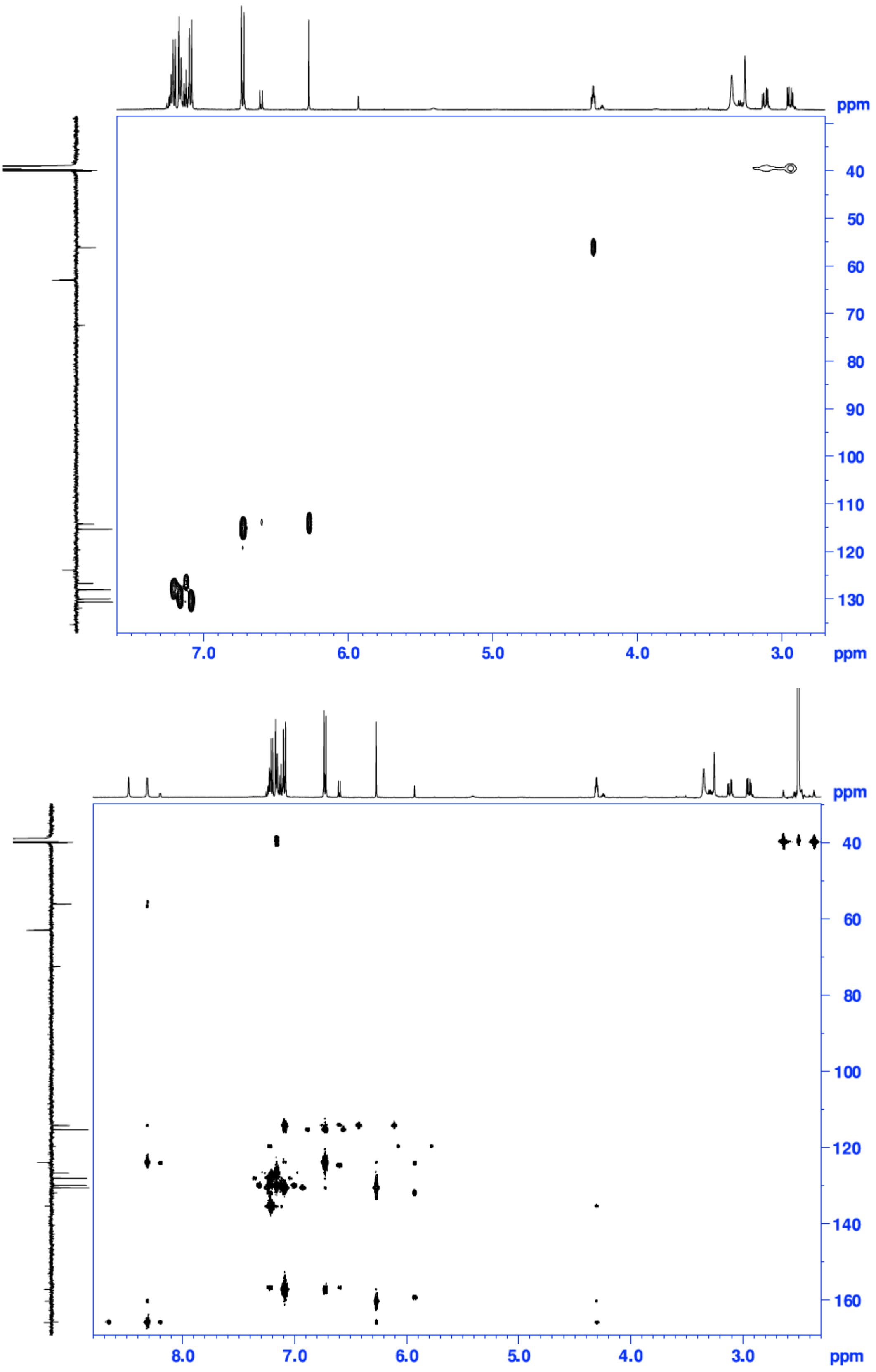

Fig. S5. 2D ^{13}C-^{1}H HSQC spectrum (top) and 2D ^{13}C-^{1}H HMBC spectrum (bottom) of cΔYF in DMSO-d_6.

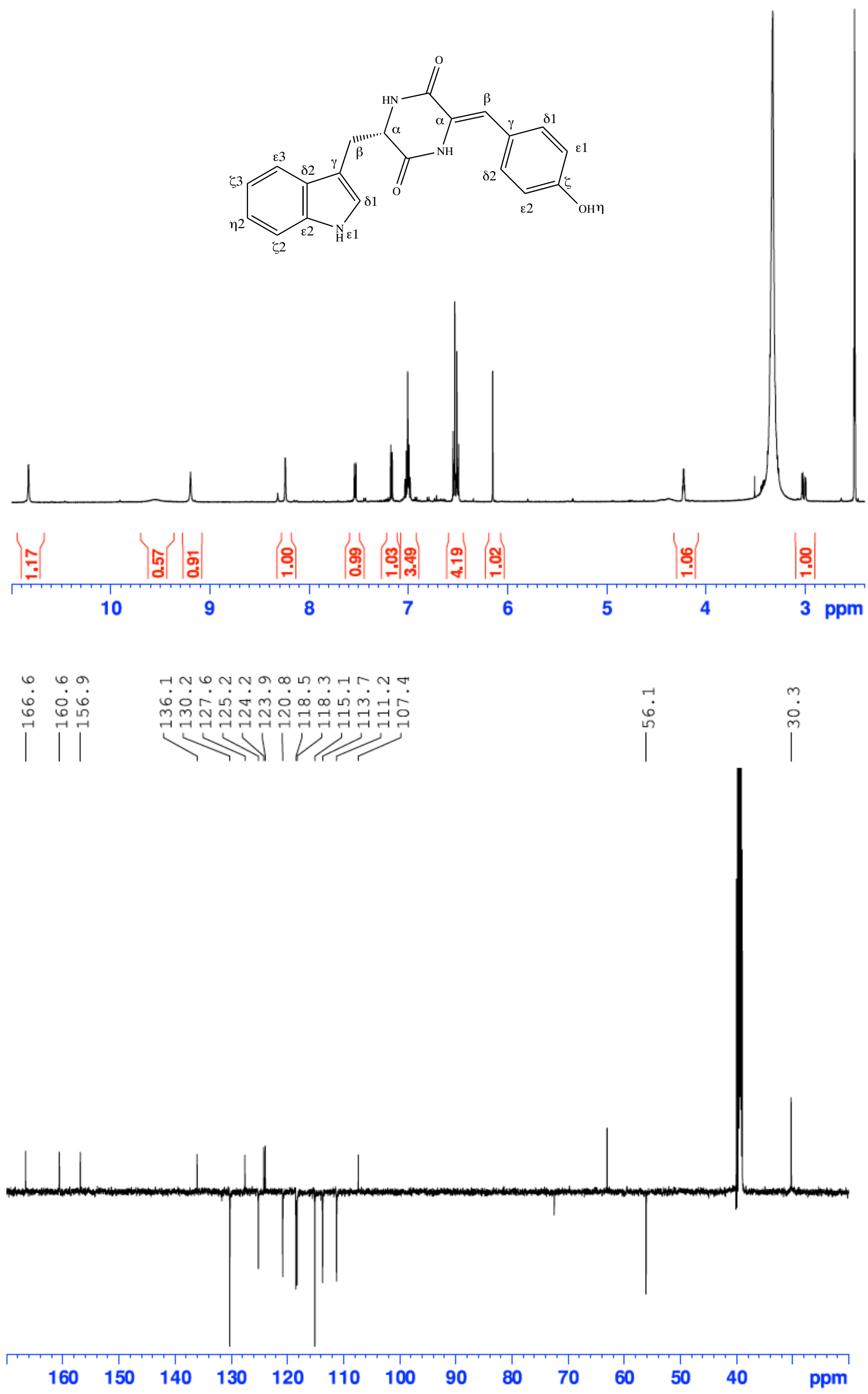

Fig. S6. 1D ^{1}H NMR spectrum (top) and 1D ^{13}C NMR DEPTQ spectrum (bottom) of cWΔY in DMSO-d_6.

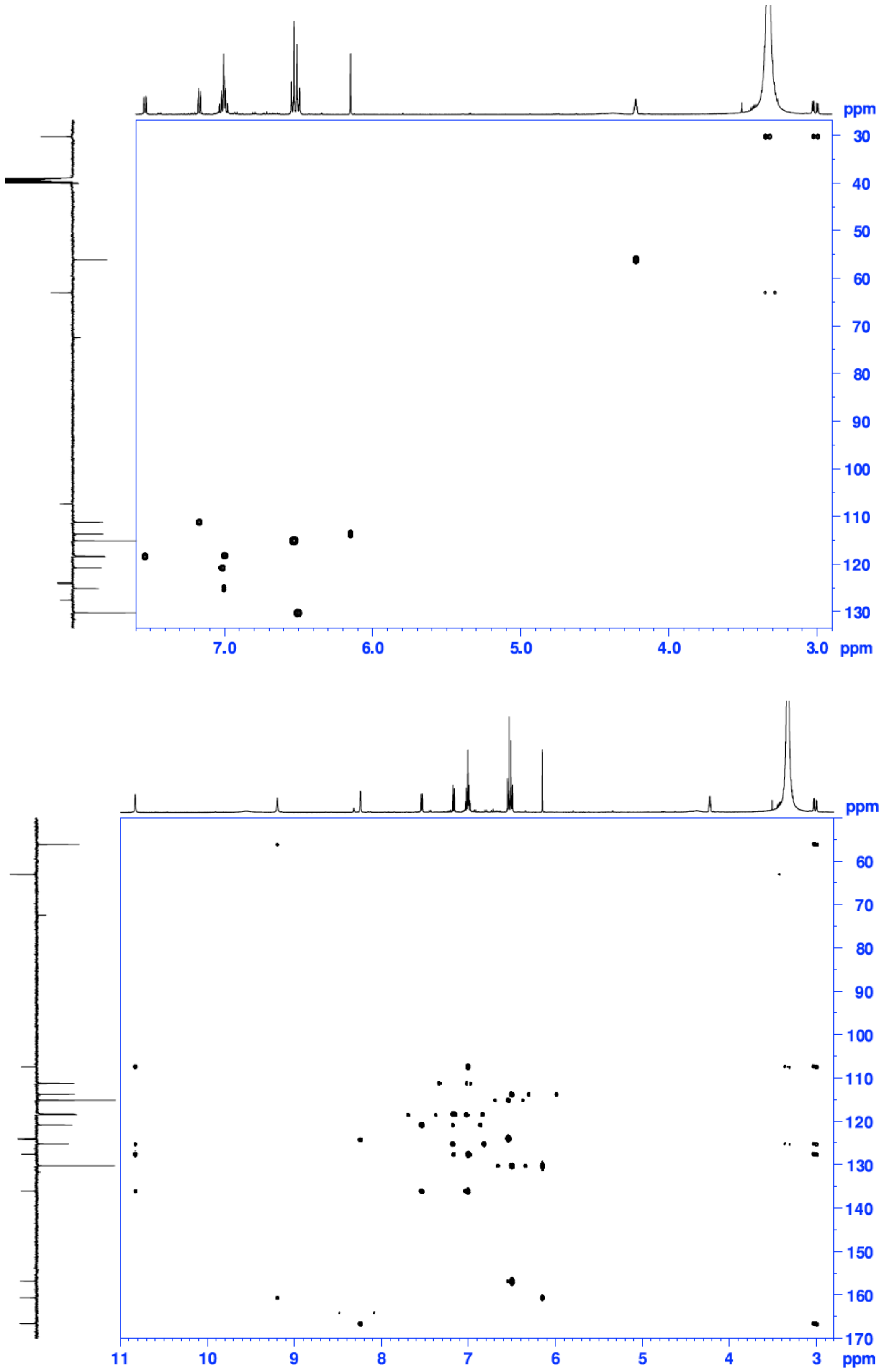

Fig. S7. 2D ^{13}C-^{1}H HSQC spectrum (top) and 2D ^{13}C-^{1}H HMBC spectrum (bottom) of cWΔY in DMSO-d_6.

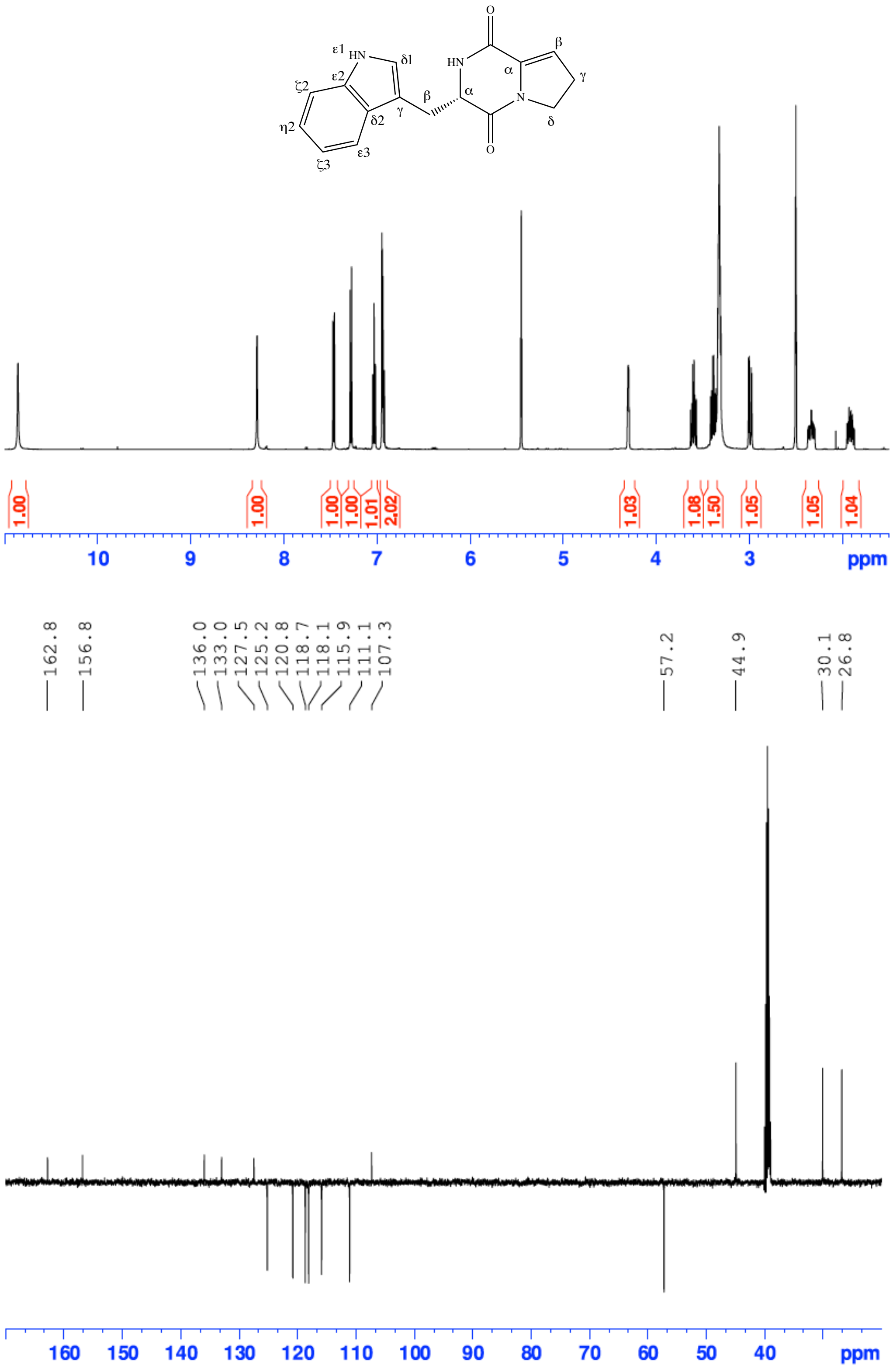

Fig. S8. 1D ^{1}H NMR spectrum (top) and 1D ^{13}C NMR DEPTQ spectrum (bottom) of cWΔP in DMSO-d_6.

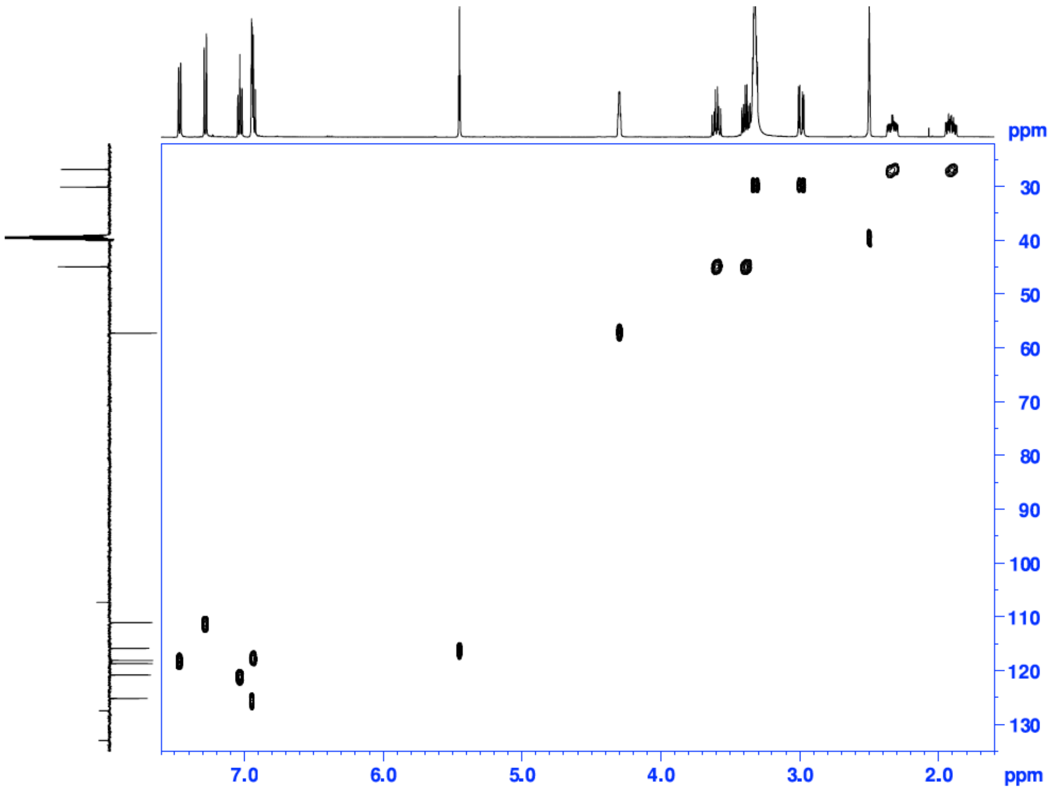

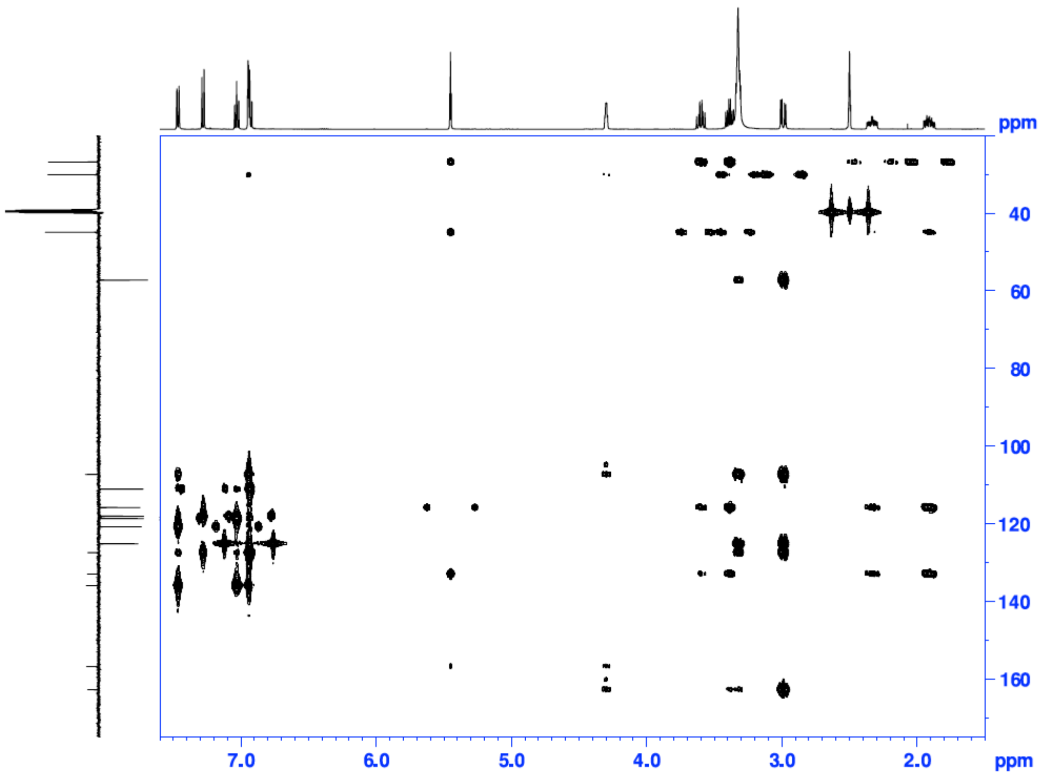

Fig. S9. 2D ^{13}C-^{1}H HSQC spectrum (top) and 2D ^{13}C-^{1}H HMBC spectrum (bottom) of cWΔP in DMSO-d_6.

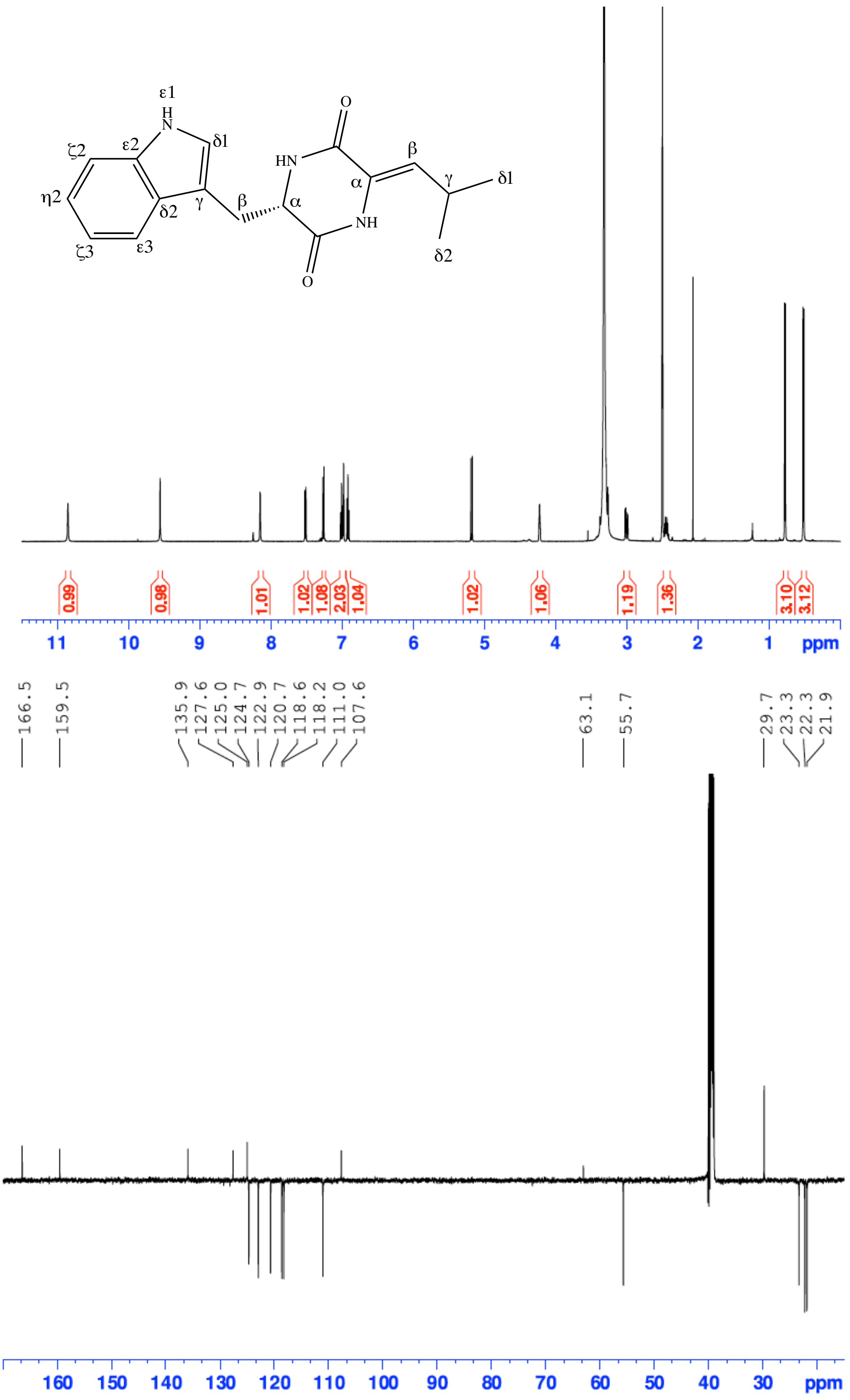

Fig. S10. 1D ^{1}H NMR spectrum (top) and 1D ^{13}C NMR DEPTQ spectrum (bottom) of cWΔL in DMSO-d_6.

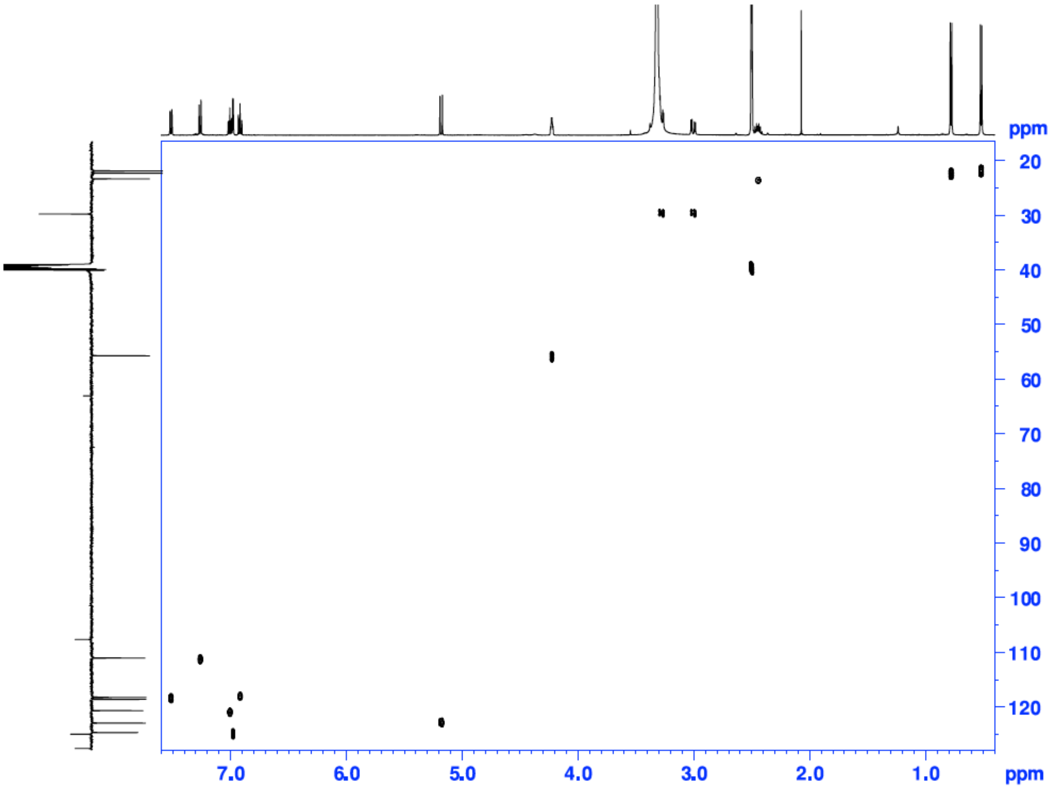

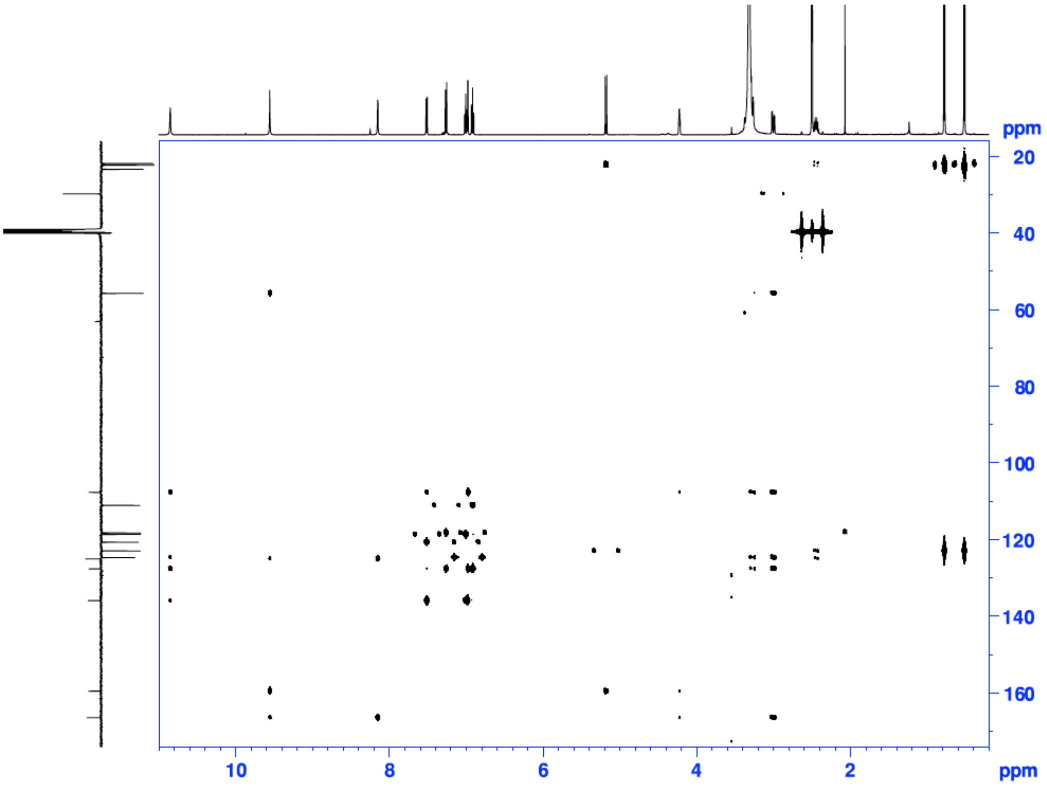

Fig. S11. 2D ^{13}C-^{1}H HSQC spectrum (top) and 2D ^{13}C-^{1}H HMBC spectrum (bottom) of cWΔL in DMSO-d_6.

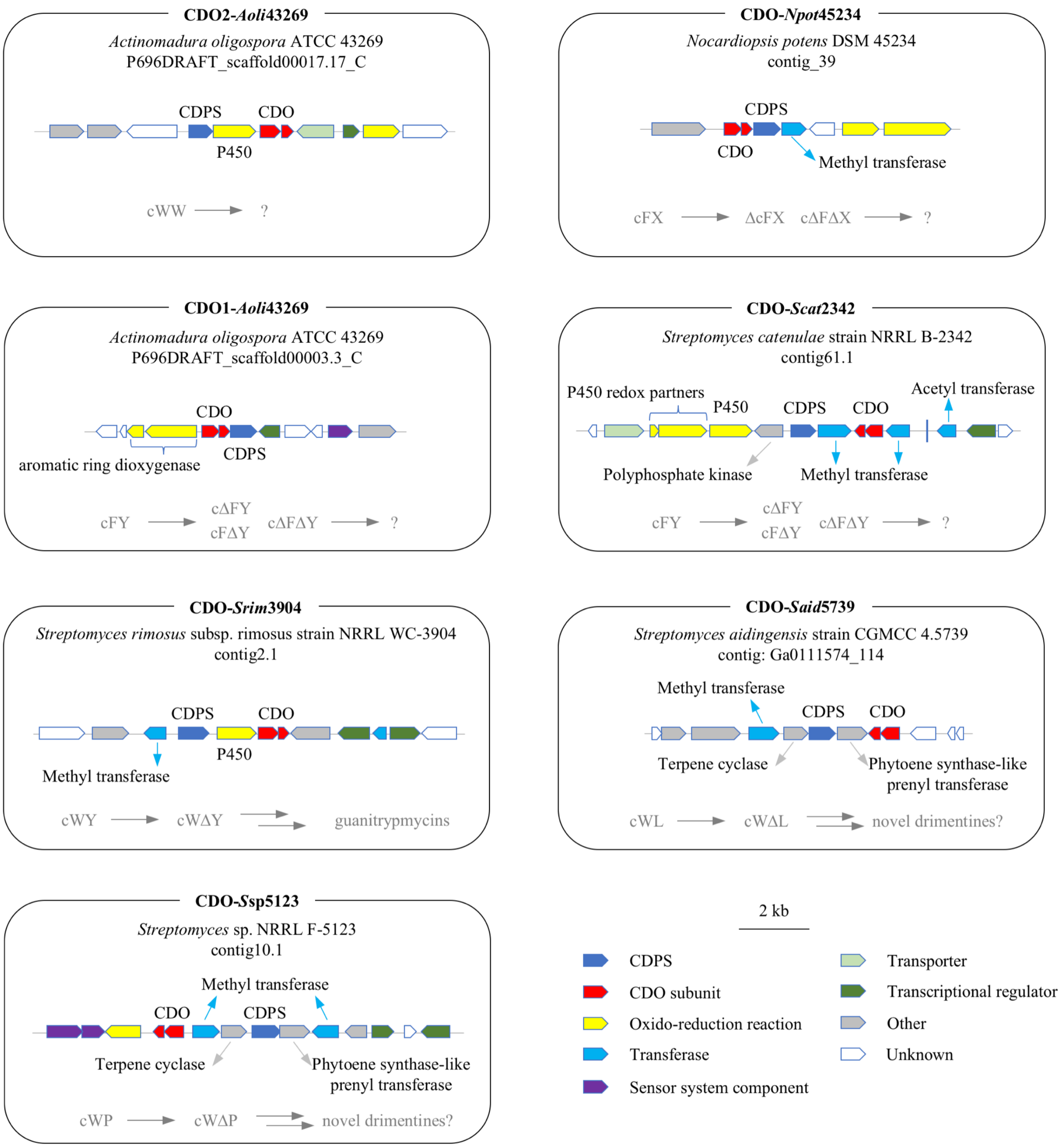

Fig. S12. Genomic environment of the CDO genes and predicted biosynthetic pathways. The genomic environments of the genes of the studied CDOs are shown. Genes are colored according to the function of the encoded protein (legend on the bottom right). For clarity, the function of proteins encoded by genes surrounding the CDPS gene is specified. For each CDO, a predicted biosynthetic pathway is proposed.
